# Liposomal Antibiotic Booster Potentiates Carbapenems for Combating NDMs‐Producing *Escherichia* *c* *oli*

**DOI:** 10.1002/advs.202304397

**Published:** 2023-11-07

**Authors:** Sixuan Wu, Yongbin Wei, Yang Wang, Zhenzhong Zhang, Dejun Liu, Shangshang Qin, Jinjin Shi, Jianzhong Shen

**Affiliations:** ^1^ School of Pharmaceutical Sciences Zhengzhou University Zhengzhou 450001 China; ^2^ Henan Key Laboratory of Targeting Therapy and Diagnosis for Critical Diseases Zhengzhou University Zhengzhou 450001 China; ^3^ Key Laboratory of Advanced Drug Preparation Technologies Ministry of Education Zhengzhou University Zhengzhou 450001 China; ^4^ School of Life Science Zhengzhou University Zhengzhou 450001 China; ^5^ Engineering Research Center for Animal Innovative Drugs and Safety Evaluation, Ministry of Education, College of Veterinary Medicine China Agricultural University Beijing 100094 China; ^6^ National Key Laboratory of Veterinary Public Health and Safety, College of Veterinary Medicine China Agricultural University Beijing 100094 China; ^7^ State Key Laboratory of Esophageal Cancer Prevention & Treatment Zhengzhou 450001 China

**Keywords:** bacteria targeting, membrane fusion liposome, New Delhi Metallo‐β‐lactamases producing Enterobacterales, ROS‐initiated drug release, Zn(II) deprivation therapy

## Abstract

Infections caused by *Enterobacterales* producing New Delhi Metallo‐β‐lactamases (NDMs), Zn(II)‐dependent enzymes hydrolyzing carbapenems, are difficult to treat. Depriving Zn(II) to inactivate NDMs is an effective solution to reverse carbapenems resistance in NDMs‐producing bacteria. However, specific Zn(II) deprivation and better bacterial outer membrane penetrability in vivo are challenges. Herein, authors present a pathogen‐primed liposomal antibiotic booster (M‐MFL@MB), facilitating drugs transportation into bacteria and removing Zn(II) from NDMs. M‐MFL@MB introduces bismuth nanoclusters (BiNCs) as a storage tank of Bi(III) for achieving ROS‐initiated Zn(II) removal. Inspired by bacteria‐specific maltodextrin transport pathway, meropenem‐loaded BiNCs are camouflaged by maltodextrin‐cloaked membrane fusion liposome to cross the bacterial envelope barrier via selectively targeting bacteria and directly outer membrane fusion. This fusion disturbs bacterial membrane homeostasis, then triggers intracellular ROS amplification, which activates Bi(III)‐mediated Zn(II) replacement and meropenem release, realizing more precise and efficient NDMs producer treatment. Benefiting from specific bacteria‐targeting, adequate drugs intracellular accumulation and self‐activation Zn(II) replacement, M‐MFL@MB rescues all mice infected by NDM producer without systemic side effects. Additionally, M‐MFL@MB decreases the bacterial outer membrane vesicles secretion, slowing down NDMs producer's transmission by over 35 times. Taken together, liposomal antibiotic booster as an efficient and safe tool provides new strategy for tackling NDMs producer‐induced infections.

## Graphical abstract

A pathogen‐primed liposomal antibiotic booster is contructed than can revive carbapenem efficiency in NDMs‐producing clinical *E. coli* isolates in vitro and in vivo, via a cascading process: pathogen‐targeting, membrane fusion‐induced physical barrier breaks, and ROS‐responsive Bi(III)‐mediated Zn(II) removal.

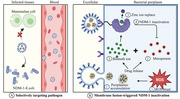

## 1 Introduction

Carbapenem‐resistant *Enterobacterales* (CRE) has been categorized as the highest priority pathogens for treatment by the World Health Organization.^[^ ^1^ ^]^ Carbapenemase is the main resistance determinant of CRE that renders bacterial resistance to nearly all β‐lactams antibiotics, including carbapenems.^[^ ^2^ ^]^ New Delhi Metallo‐β‐lactamases (NDMs) are one of the most prevalent carbapenemases and have spread over 70 countries in clinical settings since their discovery in 2009.^[^ ^3^ ^]^ In particular, the NDMs‐producing CRE can trigger multiple types of severe infection (e.g., pneumonia, septicemia, and abscesses), and kill almost half of infected in‐patients.^[^ ^4^ ^]^ NDMs is a Zn(II)‐dependent periplasmic enzyme that activates nucleophilic water to destroy the β‐lactam ring of carbapenems, thus resulting in poor clinical outcomes.^[^ ^5^ ^]^ Considering the existing antibiotic treatment failure combined with new antibiotics void,^[^ ^6^ ^]^ NDMs‐producing *Enterobacterales* leaves clinicians with few choices from the antibiotic pipeline.

To date, an economical and effective strategy for tackling NDMs producers is to revitalize existing antibiotics using antibiotic adjuvants.^[^ ^7^ ^]^ They are usually NDM inhibitors that decrease enzymatic function via kicking out the crucial Zn(II) cofactors, binding with amino acid residue of active sites, or mimicking the NDMs substrates.^[^ ^8^ ^]^ Among them, inhibitors with Zn(II) deprivation action, such as ethylenediamine‐N,N,N′,N′‐tetraacetate (EDTA), aspergillomarasmine A or bismuth (Bi(III)) compounds, have garnered more attention under their great potential in restoring the susceptibility of NDMs producers to carbapenems.^[^ ^9^ ^]^ However, such inhibitors indiscriminately displace Zn(II) from commensal bacteria and mammalian cells, therefore impairing many biological functions and triggering high off‐target toxicity in vivo application.^[^ ^9b^ ^]^ Additionally, the bacterial outer membrane has been recognized as an impermeable barrier, which hindered intracellular antibiotic and adjuvant accumulation.^[^ ^10^ ^]^ Recently, Nanotechnology has been promising for antibiotic adjuvant development due to its ability to control the loading, delivery, and release of antibiotics and to enhance the antibacterial potency.^[^ ^11^ ^]^ However, spatial and temporal control remains an unresolved obstacle for nanoparticle‐constituent adjuvants due to off‐target distributions, systemic delivery, and limited modulatory effects.

Bismuth compound has been shown to irreversibly inactive NDMs via replacing zinc ions in the NDMs active site.^[^ ^9b^ ^]^ Developing a bismuth‐based nanoadjuvant that simultaneously overcome bacterial membrane barrier and precisely inactivate NDMs provides a new opportunity to reverse carbapenems resistance in NDMs producer. However, existing bismuth‐based nanoparticles are faced with a series of shortcomings: the lower loading capacity for ions, uncontrolled release of ions, and synthetic complexity.^[^ ^12^ ^]^ Here, bismuth potassium citrate (BPC) granule, a low‐price stomach medicine (<1 China Yuan/g), was directly converted into high‐security bismuth nanoclusters (BiNCs) via UV irradiation. BiNCs was found to not only be safe for use in vivo, but also have high bismuth ion loading, ROS‐responsive dissociation, and antibiotic adsorption capabilities. These characteristics are expected to endow BiNCs with an excellent targeted‐NDMs inhibitor.

Selectively overcoming bacterial outer membrane barrier that delivers BiNCs into bacterial periplasm is another tricky problem. Liposome fusion‐based transport (LiFT) strategy features direct drugs delivery into cells via a vesicle‐cell fusion process, providing a robust tool for breaking outer membrane barrier.^[^ ^13^ ^]^ Although various membrane fusion liposome (MFL) has been developed, most of them was applied into mammalian cell transportation.^[^ ^14^ ^]^ A deeper excavation and application is urgent in bacterial transportation. Previous studies found that liposome consisting of L‐α‐phosphatidylcholine (EggPC) and cholesterol can fuse with outer membrane of Gram‐negative bacteria,^[^ ^15^ ^]^ in which abundant EggPC provides a moderate phase transition temperature to maintain the fluidity of the lipid shell similar to bacterial membrane for fusion. Additionally, rational designing a bacterial anchored‐MFL further assists in dehydrating the gap between the lipid shell and bacterial membrane, accelerating fusion process. More iomportantly, membrane fusion could change membrane permeability, and then induce the initiation of ROS‐related signal pathway,^[^ ^16^ ^]^ providing a clue for intracellular bismuth ions release. Hence, pathogen‐targeted LiFT strategy would have a significant potential to selectively overcoming bacterial outer membrane barrier.

**Scheme 1:**
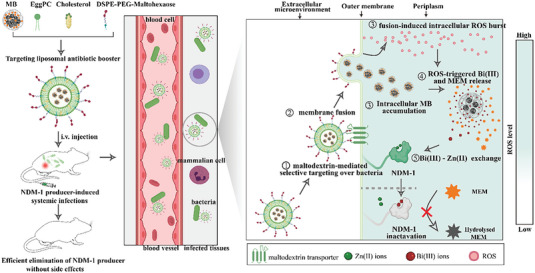
Schemes for construction of targeting liposomal antibiotic booster for targeted periplasmic NDMs inactivation via Bi(III)‐mediated Zn(II) removal. The meropenem‐loaded bismuth nanoclusters (MB) are encapsulated in the maltodextrin‐cloaked membrane fusion liposome and precisely delivered to the infectious sites with the assistance of bacteria‐specific maltodextrin transport pathway, where the liposome fuses with bacterial outer membrane, facilitating the periplasmic translocation of MB and intracellular ROS burst. Meanwhile, endogenous ROS amplification triggers the intracellular‐specific release of Bi(III) and meropenem. Released Bi(III) irreversibly inactive NDMs via displacing Zn(II) from NDMs active sites, thus protecting meropenem from hydrolyzation.

Herein, we designed a pathogen‐primed liposomal antibiotic booster for eradicating NDMs‐producing CRE via specifically inactivating periplasmic NDMs and then potentiating the efficiency of meropenem, a broad‐spectrum carbapenem (**Scheme** **1**). High‐security bismuth nanoclusters (BiNCs) act as the core for meropenem loading. The fusion‐type liposome made of L‐α‐phosphatidylcholine (EggPC), cholesterol, and DSPE‐PEG‐maltodextrin (M‐MFL) is used as targeting shell for wrapping the meropenem‐loaded BiNCs (MB). Owing to bacteria‐specific maltodextrin transport pathway, the antibiotic booster could selectively target on pathogen with the assistance of maltodextrin corona. After successfully anchoring to pathogen surface, a rapid membrane fusion behavior between liposome and bacteria broke membrane barrier for direct and efficient intracellular MB accumulation, activated intracellular ROS amplification and then triggered the intracellular‐specific release of Bi(III) and meropenem. Released Bi(III) irreversibly inhibited NDMs via displacing Zn(II) in NDMs active sites, preventing meropenem hydrolyzing. Mice infection models revealed that the antibiotic booster restores meropenem efficacy against clinical NDMs‐producing pathogen. Taken together, we developed a nanoadjuvant‐platform for re‐potentiating meropenem activity with high specificity and effectiveness, to address the severe infections caused by NDMs‐producing CRE.

## 2 Results

### 2.1 The synthesis and characterization of bismuth nanoclusters (BiNCs)

**Figure 1:**
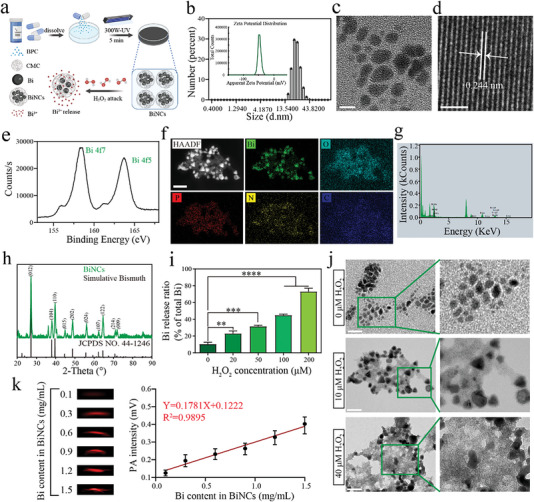
a) The scheme presenting the synthesis process of Bismuth‐Nano‐Clusters (BiNCs) and ROS‐responsive Bi(III) release from BiNCs. b) Hydrodynamic diameter distribution and zeta potential distribution (inset) obtained for BiNCs. c) Representative TEM image of BiNCs. Scale bar, 10 nm. d) A representative high‐resolution TEM image of BiNCs. Scale bar, 2.5 nm. e) XPS diffraction spectrum of BiNCs. f) STEM‐HAADF image and corresponding EDS elemental mappings of Bi, O, P, N, and C in BiNCs. g) EDS spectrum for bismuth element analysis of BiNCs. h) X‐ray diffraction (XRD) analysis of BiNCs. i) In vitro Bi(III) release from BiNCs against different levels of H_2_O_2_. j) Representative TEM images of BiNCs after 24 h incubation with different levels of H_2_O_2_. Scale bar, 100 nm. k) PA response to different concentrations of BiNCs (0.1, 0.3, 0.6, 0.9, 1.2, and 1.5 mg/mL) (inset: PA imaging of BiNCs). Data are presented as mean values ± SD, n = 3 biologically independent samples. Statistical significance was analyzed by the two‐tailed Student's t‐test. *P < 0.05, **P < 0.01, ***P < 0.001, ****P < 0.0001.

**Figure** **1**a illustrates the preparation approach of BiNCs. A clinically available stomach medicine BPC (oral bismuth potassium citrate granules) was synthesized into BiNCs via a one‐step UV irradiation method.^[^ ^17^ ^]^ The citrate and carboxymethyl cellulose (CMC) used in the synthesis are auxiliary materials in BPC, thereby avoiding the use of harmful reagents and residues of by‐products throughout the synthesis. The buffer containing BPC appeared colorless, whereas BiNCs appeared dark black (Figure S1). Dynamic light scattering revealed that the hydrodynamic diameter of BiNCs was 21.04 ± 0.99 nm, and the zeta potential was −57.8 ± 4.27 mV (Figure 1b). TEM images showed the spherical morphology of the BiNCs with a typical lattice structure and a spacing of about 0.244 nm with a homogeneous size (Figure 1c and d). The peaks at 4f7 and 4f5 in X‐ray photoelectron spectroscopy (XPS) indicated the characteristic peaks of bismuth element (Figure 1e). The TEM elemental mappings also showed the uniform distribution of Bi elements in BiNCs (Figure 1f and g). Afterward, the X‐ray powder diffraction (XRD) pattern verified that the as‐prepared BiNCs were typical bismuth phases (Figure 1h). All these results confirmed the successful preparation of BiNCs.

**Figure 2:**
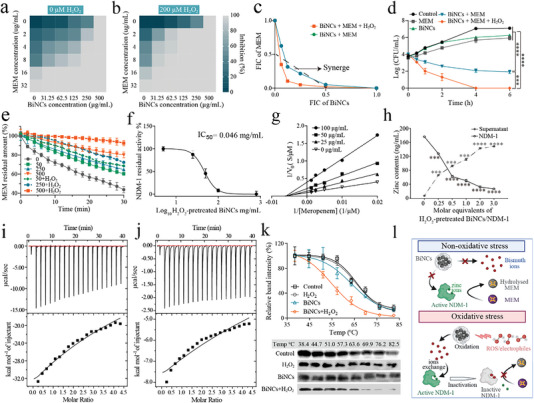
a, b) Representative heat plots of microdilution checkerboard assays for the combination of BiNCs and meropenem in the absence (a) or presence (b) of H_2_O_2_ against EC1322. c) Isobolograms of the combination of BiNCs and meropenem in the absence or presence of H_2_O_2_ against EC1322. The black dotted line shows the ideal isobole, where drugs act additively and independently. Data points below this line reveal synergism. d) Time‐kill curves for meropenem or BiNCs monotherapy, or their combination therapy in the absence or presence of H_2_O_2_ against EC1322 during 6 h incubation. The concentrations of meropenem, BiNCs, and H_2_O_2_ are used at 8 µg/mL, 125 µg/mL, and 200 µM, respectively. e) Hydrolytic effects of the BiNCs or BiNCs+H_2_O_2_ pre‐treated NDM‐1‐producing *E. coli* BL21 on meropenem (n = 3). f) Inhibition of NDM‐1 activity by Bi(III) from 200 µM H_2_O_2_‐pretreated BiNCs with IC_50_ of 0.046 mg/mL (n = 3). g) Double reciprocal plot of substrate‐dependent enzyme kinetics on inhibition of NDM‐1 activity by Bi(III) from 200 µM H_2_O_2_‐pretreated BiNCs, reflecting that Bi(III) (released from BiNCs) inhibited NDM‐1 via either a non‐competitive or an irreversible inhibition mode. h) Zn(II) content in Zn_2_‐NDM‐1 and the supernatant after being treated with different concentrations of Bi(III) from H_2_O_2_‐pretreated BiNCs by equilibrium dialysis, respectively. The metal content was determined by ICP‐MS. i, j) ITC thermograms for the binding of Bi(III) from H_2_O_2_‐pretreated BiNCs (i) or BiNCs (j) to NDM‐1. The downward peaks indicate an exothermic process. k) Cellular thermal shift assay demonstrating the binding of Bi(III) to NDM‐1 in NDM‐producing *E. coli* BL21. NDM‐1 melting temperature was shifted from 68.1° to 56.8 °C for control and BiNCs‐H_2_O_2_ combination group, respectively. The images show the western blotting result. **l** Schematic diagram of the action mechanism of BiNCs under non‐oxidative stress and oxidative stress on NDM‐1‐producing *E. coli*. Data are presented as mean values ± SD, n = 3 biologically independent samples. Statistical significance was analyzed by the two‐tailed Student's t‐test. *P < 0.05, **P < 0.01, ***P < 0.001, ****P < 0.0001.

Notably, we identified a reactive oxygen species (ROS)‐related Bi(III) release in BiNCs, which was enhanced with the increasing of H_2_O_2_ concentration (P < 0.01, Figure 1i). Morphological and hydrodynamic diameter changes of BiNCs in the presence of H_2_O_2_ also reflected ROS‐responsive disintegration of BiNCs (Figure 1j, Figure S2). BiNCs also presented excellent photoacoustic (PA) imaging properties (Figure 1k), promising to realize visualization of pathogen tracing in vivo. We further verified BiNCs can respond to ROS to resensitize NDMs producers specifically toward meropenem (MEM) in a clinical NDM‐1‐producing *E. coli* isolate EC1322 recovered from peritoneal drainage fluid (Table S1, Figure S3). Compared with the BiNCs‐MEM group, the BiNCs‐MEM‐H_2_O_2_ group exhibited synergistic growth inhibition toward EC1322 with a fractional inhibitory concentration index (FICI) of 0.375 (**Figure** **2**a‐c), and the bacterial amounts plummeted (P < 0.01) and their outgrowth was blocked throughout 4 h exposure (Figure 2d). While H_2_O_2_ itself, even at 400 µM, showed no growth inhibition and no synergistic interaction with MEM toward EC1322 (Figure S4). Additionally, BiNCs could reduce the minimal inhibitory concentration (MIC) values of MEM toward NDM‐1 producer but not NDM‐1 negative strain in the presence of H_2_O_2_ (Figure S5).

We further used an engineering *E. coli* BL21 expressing periplasmic NDM‐1 to demonstrate ROS‐powered Bi(III) release from BiNCs inactivating NDMs. Compared with single BiNCs treatment, H_2_O_2_‐pretreated BiNCs presented a more significantly obstructive effect on the hydrolysis rate of MEM in BL21 (Figure 2e). The NDM‐1 activity decreased as the 200 µM H_2_O_2_‐pretreated BiNCs concentration escalated (IC_50_ = 0.046 mg/mL), ultimately leading to the inhibition of ∼80% activities of NDM‐1 (Figure 2f and Figure S6). Enzyme kinetics analysis revealed that the apparent Vmax of NDM‐1 decreased from 10.11 to 2.53 µM/s when 200 µM H_2_O_2_ – pretreated BiNCs concentration increased from 0 to 100 µg/mL, and a typical non‐competitive or an irreversible inhibition was observed according to the relevant Line‐weaver Burk plot (Figure 2g). Next, we found that the appearance of an absorption band at 340 nm after incubating apo‐NDM‐1 (lack of Zn(II)) with H_2_O_2_‐pretreated BiNCs (Figure S7), which is characteristic for Bi–S ligand‐to‐metal charge transfer (LMCT) band, suggesting that the Bi(III) from BiNCs could bound to NDM‐1. Moreover, ICP‐MS results further revealed that the addition of increasing amounts of Bi(III) resulted in a Zn(II) removal in NDM‐1, accompanied by Bi(III) bound to NDM‐1 (Figure 2h and Figure S8). Then, the affinities of Bi(III) from H_2_O_2_‐pretreated BiNCs to NDM‐1 were closely examined by isothermal titration calorimetry (ITC), unveiling that Bi(III) rather than BiNCs have a high affinity to NDM‐1 (Figure 2i and j), and the cellular thermal shift assay further reflecting the binding of intracellular BiNCs‐released Bi(III) to NDM‐1 in intact cells (Figure 2k). Together, Bi(III) released from BiNCs in response to ROS competitively replaces the Zn(II) to bind to NDM‐1, thereby hampering the activity of NDM‐1 and leading to carbapenem resistance reversal of NDM‐1‐producing *E. coli*. (Figure 2l).

### 2.2 Fabrication and Characterization of Pathogen‐Primed Liposomal Antibiotic Booster

**Figure 3:**
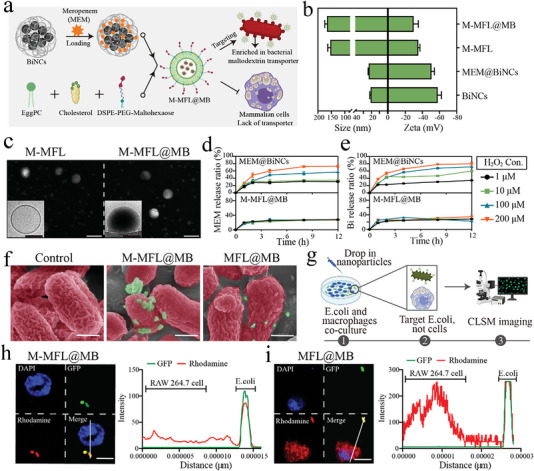
a) The schematic illustration exhibiting the preparation process of maltodextrin‐decorated membrane fusion liposome that wraps meropenem‐loaded BiNCs (M‐MFL@MB) and the highly specific bacterial targeting mechanism. b) Hydrodynamic diameter and ζ potential of BiNCs, meropenem‐loaded BiNCs (MB), maltodextrin‐decorated membrane fusion liposome (M‐MFL) and M‐MFL@MB (n = 3). c) Representative TEM and cryo‐TEM (inset) images of M‐MFL (left) and M‐MFL@MB (right). Scale bar, 100 nm. d, e) In vitro MEM (d) and Bi(III) (e) release curves of MEM@BiNCs (MB) (upper) and M‐MFL@MB (lower) in PBS containing different concentrations of H_2_O_2_ (1, 10, 100 and 200 µM), (n = 3). f) Representative pseudo‐color SEM images of EC1322 after incubation with PBS, M‐MFL@MB, and MFL@MB, respectively. Scale bar, 1 µm. g) The experimental scheme of coculture experiments for verifying the capability of maltodextrin‐mediated specifical targeting to bacteria, not mammalian cells. h, i) Confocal images of mononuclear macrophages (RAW 264.7 cells) cocultured with GFP‐expressing *E. coli* and imaged after labeling with M‐MFL@MB (h) or MFL@MB (i), respectively. Scale bar, 5 µm. The nucleus of RAW 264.7 cells, *E. coli*, and MFL were labeled with DAPI (blue), Green fluorescent protein (green), and Rhodamine (red), respectively. Plot profiles corresponding to white lines are shown on the right. Data are presented as mean values ± SD, n = 3 biologically independent samples. Statistical significance was analyzed by the two‐tailed Student's t‐test. *P < 0.05, **P < 0.01, ***P < 0.001, ****P < 0.0001.

To integrate pathogen‐targeting, precise intracellular delivery, and site‐specific release of drug capabilities, we produced pathogen‐targeting liposomal antibiotic booster (M‐MFL@MB) using a maltodextrin‐cloaked membrane‐fusion liposome (M‐MFL) as a shell and meropenem‐loaded BiNCs (MB) as a core (**Figure** **3**a). In this system, MEM loaded into BiNCs with a 50% loading yield using an optimal input of 3.2 wt% (Figure S9). Maltodextrin (MA)‐PEG‐DSPE successfully anchored to membrane‐fusion liposome (MFL) consisting of L‐α‐phosphatidylcholine (EggPC) and cholesterol via a phospholipids fusion,^[^ ^18^ ^]^ and the modification rate of MA was counted as 0.52 mg/mL. Then, MB was stored into the lumen of M‐MFL with a 29.5% loading yield (Figure S9). M‐MFL@MB (166 nm) was slightly larger than M‐MFL (154 nm) and much larger than naked MB (21 nm). M‐MFL@MB possessed an equivalent surface charge (Figure 3b), a uniform and spherical structure with a unilamellar membrane coating, which is similar to that of M‐MFL (Figure 3c). Cryo‐TEM revealed the successful cloaking of MB into M‐MFL, reflected by a deeper image lining degree from M‐MFL to M‐MFL@MB (Figure 3c). M‐MFL protected MB from disaggregation under physiological (H_2_O_2_ concentration = 10 µM) and infectious (H_2_O_2_ concentration = 100 µM) microenvironment ^[^ ^19^ ^]^ (Figure 3d and e). Once the shell is removed, MB could rapidly release MEM and Bi(III) in an H_2_O_2_ concentration‐dependent manner, 73% MEM and 80% Bi(III) were released under the 200 µM H_2_O_2_ treatment.

As a major microbial carbon source, maltodextrin (MA) is selectively internalized into bacterial cells through bacterial‐specific maltodextrin transporter, but hardly enters mammalian cells.^[^ ^20^ ^]^ As expected, MA corona endowed M‐MFL@MB with a more distinguished bacteria adhesion property than that of MFL@MB (Figure 3f). We further imaged M‐MFL@MB and MFL@MB in coculture with green fluorescence protein (GFP)‐expressing *E.coli* and mononuclear macrophages (RAW264.7 cells) (Figure 3g), and observed M‐MFL@MB targeted *E.coli* but not RAW264.7 cells (Figure 3h). The line‐scan profiles also denoted the specific co‐localization of M‐MFL@MB and bacteria. Whilst MFL@MB did not present differential targeting to *E.coli* and RAW264.7 cells (Figure 3i). Additionally, liposomal antibiotic booster also presented an excellent immune escape capability via an obvious reduction of endocytosis by monocyte‐macrophage (Figure S10a and b).

### 2.3 In Situ Bacterial Membrane Fusion, Site‐Specific Drug Transport, and Intracellular ROS Burst

**Figure 4:**
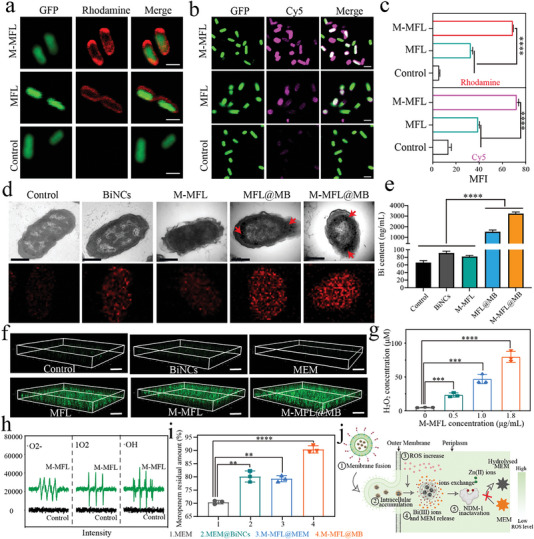
a, b) Representative confocal images visualize the membrane fusion interaction between M‐MFL or MFL with *E. coli* (a), and intracellular drug delivery of M‐MFL or MFL into *E.coli* (b). M‐MFL and MFL were labeled with fluorescent dye Rhodamine (red), Cy5 fluorescent dye (pink) was loaded into M‐MFL or MFL for substituting MB, and the *E. coli* could express GFP (green fluorescent protein, green). The control group was incubated with PBS. Scale bar, 100 nm. c) The corresponding fluorescence semi‐quantitative analysis (n = 3), shows membrane fusion and intracellular delivery efficiency of M‐MFL and MFL, respectively. d) Bio‐TEM images and the bismuth element mapping of different nanoparticles‐treated EC1322. Untreated B16‐F10 cells were used as control. Scar bar: 1 µm; Red arrows pointed to BiNCs. e) ICP‐MS analyzes the effect of different treatments on intracellular Bi(III) accumulation of *E. coli* (n = 3). f) Intracellular ROS level after being treated with different nanoparticles was monitored by detection of DCFH‐DA fluorescence intensity using confocal imaging. g) ESR spectrum of M‐MFL‐treated *E. coli*, the untreated *E.coli* was used as control. Scale bar, 30 µm. h) Determination of H_2_O_2_ amount in NDM‐1‐EC1322 after different concentrations of M‐MFL treatment (n = 3). i) Hydrolytic effects of the EC1322 on meropenem after different treatments, including MEM, MEM@BiNCs, M‐MFL@MEM and M‐MFL@MB. n = 3. j) Schematic diagram of the action mechanism of targeting liposomal antibiotic booster on NDM‐1 producers. Data are presented as mean values ± SD, n = 3 biologically independent samples. Statistical significance was analyzed by the two‐tailed Student's t‐test. *P < 0.05, **P < 0.01, ***P < 0.001, ****P < 0.0001.

M‐MFL formulation was first examined for its fusion capability with GFP‐expressing *E. coli* (**Figure** **4**a and c). The intensity of red fluorescence (MFL) in M‐MFL group was higher than that in MFL group, implying MA‐mediated pathogen targeting promotes fusion activity by accelerating bacterial adhesion. Meanwhile, flow cytometer analysis demonstrated the successful fusion (Figure S11). Compared with common liposomes constituted by soybean lecithin and cholesterol, M‐MFL exhibited a prominent membrane fusion activity due to a more similar ingredient with bacterial membrane and a higher lipid fluidity ^[^ ^21^ ^]^ (Figure S12). Förster resonance energy transfer (FRET) assay further demonstrated the specific bacterial OM fusion of M‐MFL, which was observed successfully fused with *E. coli* but hardly with platelets (Figure S13). We then demonstrated membrane fusion strategy could promote intracellular accumulation of drugs. CLSM and flow cytometry reflected that more Cy5 (substituting MB) was located inside the bacteria in M‐MFL group compared with the other groups (Figure 4b and c; Figure S14). Bio‐TEM assay showed that MB reached inside bacteria with the aid of MFL (Figure 4d). TEM elemental mappings and ICP‐MS assay further revealed the presence of abundant bismuth inside bacteria in M‐MFL@MB and MFL@MB groups (Figure 4d and e).

ROS can trigger the dissociation of MB to release MEM and Bi(III). However, the level of ROS, especially H_2_O_2_, inherent within the bacteria is less than 10 µM, only triggering little drug release (Figure 3d and e). We identified that MFL‐mediated membrane fusion strategies could endogenously trigger intracellular ROS production (Figure 4f and Figure S15‐16a). Even at 30 min, the ROS level in M‐MFL and M‐MFL@MB groups still existed steadily with no significant decreasing trend (Figure S16b), indicating that membrane fusion strategy initiated a rapid and stable ROS burst inside bacteria. The H_2_O_2_ produced inside bacteria was dependent on M‐MFL concentration, and *E.coli* incubated with 1.8 mg/mL M‐MFL produced almost 100 µM H_2_O_2_, which is adequate for MB disintegration (Figure 4g). M‐MFL could result in the generation of a variety of bacterial ROS species, including O_2_−•, ^1^O_2_, •OH, all of which have high oxidative activity for catalyzing intracellular MB disintegration. In addition, M‐MFL‐mediated membrane fusion strengthened the permeability of outer membrane and inner membrane in *E.coli* due to the impaired integrity of bacterial membrane (Figure S17a and b). Enhanced membrane permeability would trigger a change in intracellular osmotic pressure, leading to intra‐ and extracellular ions (e.g., Na^+^ and Cl^−^) homeostasis disrupted and then resulted in membrane potential depolarization, which acts as a stimulation trigger, and lastly endogenously activates intracellular ROS amplification (Figure S17c).^[^ ^16^ ^]^ Together, liposomal antibiotic booster can serve as potent NDMs inactivator to reverse MEM resistance by a cascading process: selectivity targeting over bacteria, breaking membrane barrier, specifically accumulating intracellular drugs, endogenously activating ROS amplification for intracellular‐specific release of Bi(III) and MEM (Figure 4i and j). Thus, M‐MFL@MB prevents MEM from hydrolyzing in NDM‐1 producer more effectively, compared with MEM, MB and M‐MFL@MEM (P < 0.01, Figure S18).

### 2.4 Liposomal Antibiotic Booster Resensitizes NDM‐1‐Producing *E. Coli* to MEM In Vitro

**Figure 5:**
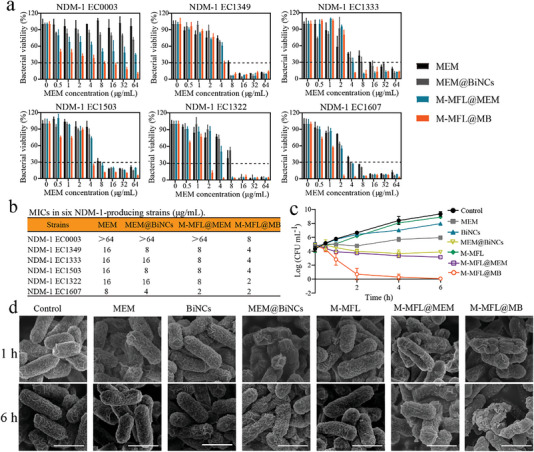
a) Measurement of bacterial colony‐forming units, obtained from six clinical isolates of NDM‐1‐producing *E. coli* treated with different concentrations of meropenem (MEM), MB, M‐MFL@MEM and M‐MFL@MB, respectively (n = 6). b) MIC of MEM, MB, M‐MFL@MEM and M‐MFL@MB in six clinical isolates of NDM‐1‐positive *E. coli* (n = 6). c) Time‐kill curves for MEM, BiNCs, M‐MFL, MB, M‐MFL@MEM, and M‐MFL@MB against EC1322 during 6 h incubation, respectively (n = 3). The concentrations of MEM were 8 µg/mL in those groups. The concentrations of BiNCs and M‐MFL were about 53.33 and 177.77 µg/mL, respectively. d) Representative SEM images of EC1322 after treatment with different nanoparticles for 1 h and 6 h, respectively. Scale bar, 0.5 µm. Data are presented as mean values ± SD, n = 3 biologically independent samples. Statistical significance was analyzed by the two‐tailed Student's t‐test. *P < 0.05, **P < 0.01, ***P < 0.001, ****P < 0.0001.

The bactericide of targeting liposomal antibiotic booster was evaluated using six NDM‐1‐producing clinical *E. coli* isolates (Table S1, Figure S3). These strains showed much higher MICs (> 64 to 2 µg/mL) in individual MEM, BiNCs, MB, M‐MFL and M‐MFL@MEM groups, respectively (**Figure** **5**a and Figure S19). Whilst M‐MFL@MB exhibited a concentrate‐dependent inhibitory effect (8 to 2 µg/mL) on all tested isolates (Figure 5b), indicating that M‐MFL@MB could reverse MEM resistance in NDM producers. Time‐dependent killing of EC1322 showed M‐MFL@MB had excellent bactericidal activity against NDMs producer (Figure 5c), also reflected by SEM results (Figure 5d). Additionally, M‐MFL@MB destructed more exhaustive bacterial structure than other groups (Figure 5d). These results suggested that targeting liposomal antibiotic boosters could resensitize NDM‐1‐producing *E. coli* to MEM in vitro. Notably, the antibiotic booster also showed synergies with ceftazidime (β‐lactam antibiotics) against NDM‐1‐producing *E. coli* isolates, but not with ciprofloxacin (quinolones) and colistin (polypeptide antibiotics), as shown in Fig. S20 and S21. The result implied that antibiotic booster mainly induces NDM‐1 inactivation, lastly reversing NDMs producer resistance against β‐lactam antibiotics.

### 2.5 Liposomal Antibiotic Booster Targets Bacterial Infectious Sites and Restores Meropenem Efficacy In Vivo

**Figure 6:**
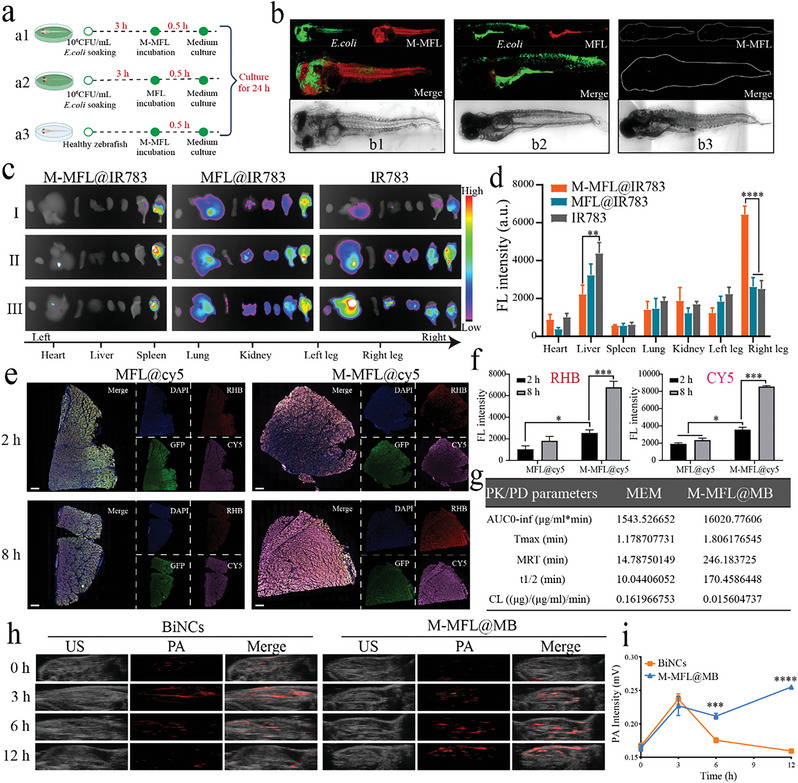
a) Experimental scheme of the bacterial‐infected zebrafish model for demonstrating maltodextrin‐mediated adhesion ability. b) Lateral views of the whole bacteria‐infected zebrafish after DiI‐labeled M‐MFL treatment (b1). The bacterial‐infected zebrafish incubated with the DiI‐ labeled MFLipo treatment (b2) and the healthy zebrafish incubated with DiI‐labeled M‐MFL (b3) were used as the control. c, d) Ex‐vivo tissue NIR FL images I and fluorescence semi‐quantitative analysis (d) of mice model of dual infection with LPS and *E. coli* at 22 h post‐injection with M‐MFL@IR783, MFL@IR783, or IR783, respectively (n = 3). e, f) Representative CLSM images I and fluorescence semi‐quantitative analysis (f) of MFL, M‐MFL distribution at different time points after intravenous injection in the *E. coli* infected lung tissues, respectively. The nanoliposome was stained with rhodamine (red). The encapsulated drugs were replaced by cy5 (pink). The nucleus of lung tissues was stained with DAPI (blue). Scar bar, 100 µm. h, i) PA imaging (h) and PA response value (i) of *E. coli*‐infected mice model at different time points after intravenous injection with BiNCs and M‐MFL@MB, respectively. Data are presented as mean values ± SD, n = 3 biologically independent samples. Statistical significance was analyzed by the two‐tailed Student's t‐test. *P < 0.05, **P < 0.01, ***P < 0.001, ****P < 0.0001.

The targeting capability of M‐MFL@MB was evaluated in zebrafish infected with GFP‐expressing *E. coli* (**Figure** **6**a). Compared with MFL group, M‐MFL group showed a more pronounced red fluorescence (RhB) trapped inside the bacteria with green fluorescence in zebrafish, demonstrating the M‐MFL can effectively recognize and adhere to the bacteria under the assist of MA (Figure 6b1 and b2). Whilst M‐MFL cannot retained inside the healthy zebrafish (Figure 6b3). Moreover, MA crown could distinguish bacterial infection from inflammation in a mouse bacterial and inflammatory co‐infection model. IVIS imaging showed more M‐MFL could accumulate effectively and specifically into the bacterial infectious tissue rather than inflammatory tissue (Figure S22a‐e), and ex vivo tissues imaging further verified the MA‐mediated specific bacterial targeting ability of M‐MFL (Figure 6c and d). The mouse lung infection model further revealed that M‐MFL could facilitate drugs accumulation at infectious sites, as RhB‐labelled M‐MFL@Cy5, not MFL@Cy5, could be gradually accumulated into infected lung, and presented an excellent co‐location with bacteria in infected tissues (Figure 6e and f).

Pharmacokinetic analysis revealed higher concentrations of M‐MFL@MB (86.32‐15.67 µg/mL) in plasma compared with the free MEM (81.85‐5.25 µg/mL) at the indicating time points (Figure S23). Compared with free MEM, the area‐under‐the‐curve (AUC0–∞), half‐life time (t1/2), and mean residence time (MRT0–∞) were distinctly improved in M‐MFL@MB‐injected mice. The blood clearance (CL) rate in M‐MFL@MB group has dropped nearly 10 times compared with MEM group (Figure 6g). An excellent photoacoustic imaging ability of M‐MFL@MB was also observed similar to BiNCs. The good PA imaging property jointing with specific targeting ability over bacterial infectious sites endowed M‐MFL@MB with a prominent diagnostic performance (Figure 6h and i).

**Figure 7:**
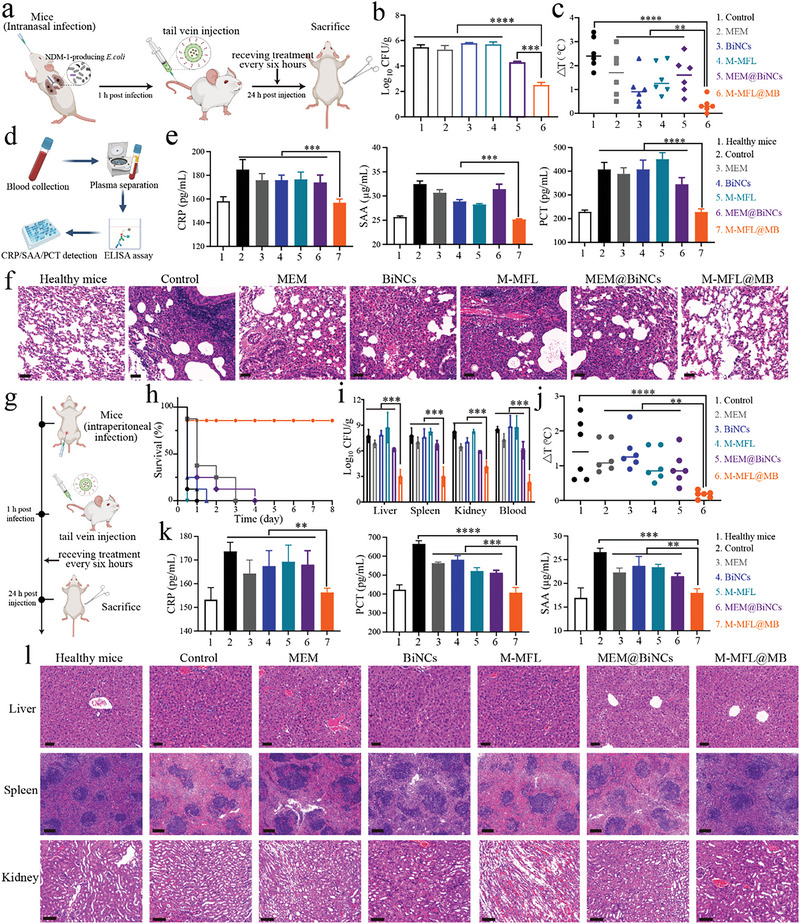
a) Schematic diagram of the infection, treatment used in mice with pneumonia. b, c) Bacterial loads (b) and mouse body temperature changes (c) in the pneumonia mice model after different nanoformulations treatment (n = 6). d) Experimental roadmap for detecting inflammation‐related indicators (CRP, SAA and PCT) of mice with pneumonia. e) CRP, SAA and PCT level in the pneumonia mice model after different treatments (n = 6). f) HE staining of infected lung tissues in the pneumonia mice model after different treatments. Scar bar, 100 µm. g) Schematic diagram of the infection, treatment used in mice with sepsis. h, i, j) Survival curves (h), bacterial loads (i) and body temperature changes (j) in the sepsis mice model after different treatments, n = 6. k) CRP, SAA and PCT level in the sepsis mice model after different treatments (n = 6). l) HE staining of infected tissues in the sepsis mice model subjected to different treatments. Scar bar, 100 µm. Data are presented as mean values ± SD, n = 3 biologically independent samples. Statistical significance was analyzed by the two‐tailed Student's t‐test. *P < 0.05, **P < 0.01, ***P < 0.001, ****P < 0.0001.

**Figure 8:**
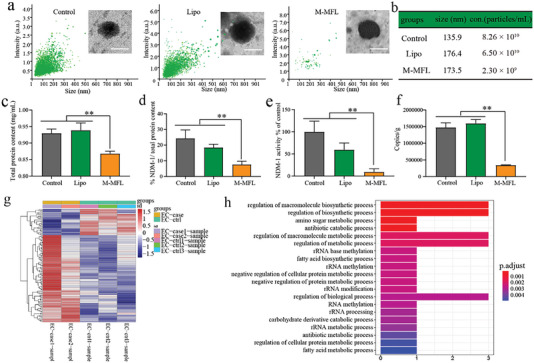
a) an NTA analysis and representative TEM images (inset) of *E. coli* OMVs extracted from *E. coli* incubated with Lipo and M‐MFL, respectively. The OMVs extracted from untreated *E. coli* were used as control. Scar bar, 100 nm. b) Statistics of size and concentration of *E. coli* OMVs extracted from *E. coli* incubated with different nanoparticles. b, c, d) the measurement of total protein content (b, n = 3), NDM‐1 protein content (c, n = 3) and enzymatic activity of NDM‐1 (d, n = 3) of *E. coli* OMVs extracted from *E. coli* incubated with Lipo and M‐MFL, respectively. The OMVs extracted from untreated *E. coli* were used as control. e) Absolute copy number of *bla*_NDM‐1_ gene in *E. coli* OMVs extracted from *E. coli* incubated with Lipo and M‐MFL, respectively. The OMVs extracted from untreated *E. coli* were used as control. n = 3. f) The MIC of EC15922 after incubation with different *E. coli* OMVs (n = 6). g) Clustering heat map of differentially expressed genes (DEGs) between M‐MFL‐treated *E. coli* and untreated *E. coli*. The abscissa is the sample name, and the ordinate is the normalized value of the DEGs. The redder the color, the higher the expression level, and the bluer the expression level, the lower the expression level. h) KEGG down‐regulated pathway enrichment analysis of differentially expressed genes between M‐MFL and control treatment group. Data are presented as mean values ± SD, n = 3 biologically independent samples. Statistical significance was analyzed by the two‐tailed Student's t‐test. *P < 0.05, **P < 0.01, ***P < 0.001, ****P < 0.0001.

A panel of biosafety evaluation assays supported the excellent biocompatibility and low toxicity profiles of M‐MFL@MB (Figures S24–S27). The bismuth metabolism in vivo showed that the liver and spleen are dominant organs for its accumulation and metabolism, which may be mainly due to RES absorption (Figure S28).^[^ ^12b^ ^]^ A mouse lung infection model (**Figure** **7**a) revealed a remarkable decrease of clinical EC1322 isolates on lung tissue in M‐MFL@MB group (**Figure** **8**b). The body temperature, severed as an important indicator of pneumonia recovery,^[^ ^22^ ^]^ had the least change in M‐MFL@MB group (Figure 7c). Additionally, the obvious decrease trends of three bacterial infectious biomarkers, c‐reactive protein (CRP), serum amyloid A (SAA) and procalcitonin (PCT) were observed after treatment in M‐MFL@MB groups, reflecting the pneumonia control after therapy (Figure 7d and e). Hematoxylin‐Eosin (HE) staining further confirmed the recovery of pneumonia mice after M‐MFL@MB treatment (Figure 7f).

We further investigated the systemic therapeutic effect of M‐MFL@MB on a murine sepsis model prepared by the intraperitoneal injection of EC1322 (10^6^ CFU) (Figure 7g). Neither MEM nor other treatments protected any of the septic mice from death within 96 hours, while 80% septic mice were rescued in M‐MFL@MB group (Figure 7h). The bacterial load in the liver, spleen, kidney, and blood in M‐MFL@MB‐treated septic mice was reduced compared with that in the other therapeutic formulation groups. Particularly, M‐MFL@MB treatment resulted in nearly 10^4^, 10^4^, 10^3,^ and 10^5^ bacterial reductions in the liver, spleen, kidney, and blood compared with MEM group, respectively (P < 0.001) (Figure 7i). The stable body temperature and the decreased trends of clinically related infectious indicators were also observed after M‐MLF@MB treatment (Figure 7j and k). Together with the dramatic decrease in leukocyte infiltration and relatively normal organizational structure in M‐MFL@MB group compared with other treatments (Figure 8l), the in vitro antimicrobial activity of M‐MFL@MB could be converted into in vivo efficacy.

### 2.6 Liposomal Antibiotic Booster Limits Resistance Dissemination by Blocking OMVs Secretion

Bacterial outer membrane vesicles (OMVs) can act as vehicle for transferring NDM‐1 protein and *bla*_NDM‐1_ gene among different pathogens, resulting in resistance spreading.^[^ ^23^ ^]^ Prevention of OMVs secretion is therefore a feasible strategy for blocking resistance spreading. Inspired by membrane fusion as an effective way of membrane perturbation via fusing with outer membrane,^[^ ^15^ ^]^ we evaluated the interference role of M‐MFL‐induced membrane fusion behavior on OMVs secretion in a clinical NDM‐1 producing *E. coli* isolate (EC1429). The morphology of OMVs upon different treatments was not affected (Figure 8a). However, the number of OMVs in M‐MFL group was significantly decreased (Figure 8a–c). Compared with other groups, an obvious reduction of NDM‐1 in total protein was found in M‐MFL group (Figure 8d). The decrease in enzymatic activity of NDM‐1 also paralleled the decrease in NDM‐1 levels (Figure 8e). M‐MFL‐treated *E. coli* secret the OMVs containing the lower level of *bla*_NDM‐1_ gene abundance, whereas untreated and lipo‐treated *E. coli* had a negligible impact on the level of *bla*_NDM‐1_ inside OMVs (Figure 8f and Figure S29). RNA‐seq further revealed the effects of M‐MFL on the RNA expression in EC1429, and 108 differentially expressed genes (DEG) between the control sample and M‐MFL treated sample were verified (Figure 8g). The M‐MFL treatment significantly down‐regulated the biosynthetic and metabolic process of EC1349 (Figure 8h), including fatty acid biosynthetic process, rRNA methylation, carbohydrate derivative catabolic process and regulation of cellular protein metabolic process, which are closely related to the composition and secretion of OMVs.^[^ ^24^ ^]^ Above all, liposomal antibiotic booster plays a positive role in blocking resistance dissemination by decreasing both the NDM‐1 production and OMV secretion.

## 3 Discussion

Novel treatments against NDMs‐positive *Enterobacterales*, which exhibited multidrug‐resistant (MDR) or extensively drug‐resistant (XDR) profiles, are urgently needed in clinical practice since colistin and tigecycline were largely compromised due to the emerging and spreading of mobile colistin resistance gene *mcr‐1* and tigecycline resistance genes *tet*(X3) and *tet*(X4).^[^ ^25^ ^]^ Given that few antimicrobials against Gram‐negative pathogens for entering clinical trials, developing NDMs inhibitors to restore carbapenem activity is a promising strategy. However, the structural diversity in active sites of metallo‐β‐lactamases (MBLs) restricted the development of effective MBL inhibitors.^[^ ^26^ ^]^ As a common active site shared by different types of MBLs (e.g., NDM, IMP, and VIM), Zn(II) is an ideal target for MBL inhibitors. Based on Zn(II)‐binding inhibition mode, two types of MBL inhibitors, metal‐depriving compounds (AMA) and metal ion [Bi(III)] replacing compounds, have been demonstrated.^[^ ^9^, ^27^ ^]^ In the development of clinically useful inhibitors, however, these small‐molecule MBL inhibitors are faced with challenges of metalloenzyme selectivity in vivo and efficient intracellular accumulation.^[^ ^28^ ^]^ Recently, nanomaterial‐based therapeutics with unique advantages in antibacterial effects attracted more attention.^[^ ^29^ ^]^ It can be served as drug carrier to augment the potency of antibiotics or be used instead of antibiotics to exert entirely new antibacterial actions. Herein, we combined the high NDMs inhibition efficacy of Bi(III) and the advantages of pathogen targeting and membrane barrier breakthrough of nanomaterial‐based therapeutics to design and construct M‐MFL@MB, a liposomal antibiotic booster that can effectively target pathogens and achieve the intracellular co‐delivery of meropenem and Bi(III) through membrane fusion.

Lipopolysaccharide‐coated outer membrane of Gram‐negative bacteria was considered as a barrier for compounds crossing. Small molecular MBL inhibitors traverse outer membrane mainly through narrow *β*‐barrel porins (eg, OmpF and OmpC).^[^ ^30^ ^]^ Thus, the cellular accumulation of NDMs inhibitors is an important factor affecting their effectiveness in rescuing carbapenem activity.^[^ ^28^ ^]^ To address this challenge, we chose liposomes to carry adjuvants and antibiotics to break through the out‐membrane barrier via membrane fusion. To date, abundant advantages of commercial liposomes such as mature production and high biocompatibility have been described,^[^ ^31^ ^]^ and these liposome has been successfully used in clinical or preclinical practice for improving the delivery efficiency of antibiotics or antitumor drugs to disease sites.^[^ ^32^ ^]^ However, understanding of the capability of bacterial outer membrane penetrability of liposome remains limited. Here, we introduced the targeting membrane fusion liposomes that can effectively reduce in vivo off‐target toxicity of inhibitors and help inhibitors and antibiotics cross the barrier of bacterial outer membrane. Moreover, we found, for the first time, that liposome‐mediated membrane fusion could endogenously activate bacterial intracellular ROS amplification, providing a self‐activated “key” for Bi(III) release into bacterial periplasm, leading to an in‐situ Bi(III)‐mediated Zn(II) deprivation. Additionally, membrane fusion strategy also slowed down NDMs‐related resistance dissemination by decreasing the secretion of bacterial OMVs. Taken together, this strategy improves the effect of Bi(III) on accurately inactivating NDM‐1 in vivo and reduces its off‐target toxicity, therefore is a promising approach for its possible application in clinical settings.

We acknowledged few limitations existed in this study. First, the visible light‐mediated decomposition of BiNCs needs to be further addressed. Second, more accurate ROS burst mechanisms originating from membrane fusion liposome need to be further elucidated. Third, more evaluations in larger preclinical such as nonhuman primates, are needed to be conducted to advance clinical translation. Nevertheless, all ingredients in the M‐MFL@MB, including liposome, maltodextrin and gastric drug, were FDA‐approved and easily obtained at the kilogram level, which is expected to promote the clinical translation of antibiotic booster.

In summary, we developed a pathogen‐primed liposomal antibiotic booster, M‐MFL@MB (maltodextrin‐cloaked membrane fusion liposome‐encapsulated meropenem‐loaded BiNCs) for reviving carbapenem efficiency in NDMs‐producing clinical *E. coli* isolates in vitro and in vivo. M‐MFL@MB decreased the mortality of infected mice via its pathogen‐targeting, physical barrier breaks, and ROS‐responsive Bi(III)‐mediated Zn(II) removal. Additionally, membrane fusion strategy mediated by M‐MFL decreased the secretion of bacterial OMVs and slowed down the resistance spreading. Our work offered a potential nano‐adjuvant platform for repurposing carbapenems potency and curing NDMs‐producer infections.

## Conflict of Interest

The authors declare no conflict of interest.

## Author Contributions

S.W., Y.W., and Y.W., contributed equally to this work. S.X.W., J.J.S. and S.S.Q. performed conceptualization, S.X.W. and Y.B.W. performed methodology, Y.B.W. and J.J.S. performed investigation, S.X.W., D.J.L., and Y.B.W. performed visualization, J.J.S., S.S.Q., Y.W., and D.J.L. performed funding acquisition, Z.Z.Z., J.Z.S., and S.S.Q. performed supervision, S.X.W., Y.B.W., and Y.M.W., wrote the original draft, S.X.W., Y.W., J.J.S., S.S.Q., and J.Z.S. wrote, reviewed and edited the manuscripts.

## Supporting information



Supporting Information

## Data Availability

Research data are not shared.

## References

[1] a) Q. Wang , X. Wang , J. Wang , P. Ouyang , C. Jin , R. Wang , Y. Zhang , L. Jin , H. Chen , Z. Wang , F. Zhang , B. Cao , L. Xie , K. Liao , B. Gu , C. Yang , Z. Liu , X. Ma , L. Jin , X. Zhang , S. Man , W. Li , F. Pei , X. Xu , Y. Jin , P. Ji , H. Wang , Clin. infect. Dis. 2018, 67, S196; 30423057 10.1093/cid/ciy660

[2] P. Nordmann , T. Naas , L. Poirel , Emerg. Infect. Dis. 2011, 17, 1791. 22000347 10.3201/eid1710.110655 PMC3310682

[3] a) W. Wu , Y. Feng , G. Tang , F. Qiao , A. McNally , Z. Zong , Clin. Microbiol. Rev. 2019, 32, e00115; 30700432 10.1128/CMR.00115-18 PMC6431124

[4] R. K. Srivastava , R. I. Ichhpujani , S. Khare , A. Rai , L. S. Chauhan , Indian J Med Res 2011, 133, 458. 21623026 PMC3121272

[5] G. Zhang , Q. Hao , FASEB J. 2011, 25, 2574. 21507902 10.1096/fj.11-184036

[6] K. Iskandar , J. Murugaiyan , D. Hammoudi Halat , S. E. Hage , V. Chibabhai , S. Adukkadukkam , C. Roques , L. Molinier , P. Salameh , M. Van Dongen , Antibiotics 2022, 11, 182. 35203785 10.3390/antibiotics11020182 PMC8868473

[7] B. Ma , C. Fang , L. Lu , M. Wang , X. Xue , Y. Zhou , M. Li , Y. Hu , X. Luo , Z. Hou , Nat. Commun. 2019, 10, 3517. 31388008 10.1038/s41467-019-11503-3 PMC6684654

[8] a) C. L. Tooke , P. Hinchliffe , E. C. Bragginton , C. K. Colenso , V. H. A. Hirvonen , Y. Takebayashi , J. Spencer , J. Mol. Biol. 2019, 431, 3472; 30959050 10.1016/j.jmb.2019.04.002 PMC6723624

[9] a) A. M. King , S. A. Reid‐Yu , W. Wang , D. T. King , G. De Pascale , N. C. Strynadka , T. R. Walsh , B. K. Coombes , G. D. Wright , Nature 2014, 510, 503; 24965651 10.1038/nature13445 PMC4981499

[10] E. R. Rojas , G. Billings , P. D. Odermatt , G. K. Auer , L. Zhu , A. Miguel , F. Chang , D. B. Weibel , J. A. Theriot , K. C. Huang , Nature 2018, 559, 617. 30022160 10.1038/s41586-018-0344-3 PMC6089221

[11] a) Y. Wang , Y. Yang , Y. Shi , H. Song , C. Yu , Adv. Mater. 2020, 32, 1904106; 10.1002/adma.201904106 31799752

[12] a) O. Rabin , J. Manuel Perez , J. Grimm , G. Wojtkiewicz , R. Weissleder , Nat. Mater. 2006, 5, 118; 16444262 10.1038/nmat1571

[13] M. Lin , Y. Chen , S. Zhao , R. Tang , Z. Nie , H. Xing , Angew. Chem., Int. Ed. 2021, 61. 10.1002/anie.202111647 34637590

[14] a) Y. Chen , T. Wu , S. Xie , Y. Bai , H. Xing , Sci. Adv., 9, eadg2583; 10.1126/sciadv.adg2583 PMC10171822 37163595

[15] S. Thamphiwatana , W. Gao , M. Obonyo , L. Zhang , Bio. Sci. 2014, 111, 17600. 10.1073/pnas.1418230111 PMC4267350 25422427

[16] S. Shabala , L. Shabala , Biomol. Concepts 2011, 2, 407. 25962045 10.1515/BMC.2011.032

[17] Y. Xuan , X. Q. Yang , Z. Y. Song , R. Y. Zhang , D. H. Zhao , X. L. Hou , X. L. Song , B. Liu , Y. D. Zhao , W. Chen , Adv. Funct. Mater. 2019, 29.

[18] X. Pang , Q. Xiao , Y. Cheng , E. Ren , L. Lian , Y. Zhang , H. Gao , X. Wang , W. Leung , X. Chen , G. Liu , C. Xu , ACS Nano 2019, 13, 2427. 30657302 10.1021/acsnano.8b09336

[19] Y. S. Raval , A. Mohamed , H. M. Zmuda , R. Patel , H. Beyenal , Glob Chall 2019, 3, 1800101. 31218078 10.1002/gch2.201800101 PMC6551415

[20] X. Ning , S. Lee , Z. Wang , D. Kim , B. Stubblefield , E. Gilbert , N. Murthy , Nat. Mater. 2011, 10, 602. 21765397 10.1038/nmat3074 PMC6443089

[21] a) J. Yang , A. Bahreman , G. Daudey , J. Bussmann , R. C. Olsthoorn , A. Kros , ACS Cent. Sci. 2016, 2, 621; 27725960 10.1021/acscentsci.6b00172 PMC5043431

[22] D. Peres Bota , F. Lopes Ferreira , C. Mélot , J. L. Vincent , Intensive care medicine 2004, 30, 811. 15127194 10.1007/s00134-004-2166-z

[23] M. M. B. Martinez , R. A. Bonomo , A. J. Vila , P. C. Maffia , L. J. Gonzalez , mBio 2021, 12, e0183621. 34579567 10.1128/mBio.01836-21 PMC8546644

[24] a) P. Han , A. Lai , C. Salomon , S. Ivanovski , Int. J. Mol. Sci. 2020, 21, 5273; 32722322 10.3390/ijms21155273 PMC7432462

[25] a) Y.‐Y. Liu , Y. Wang , T. R. Walsh , L.‐X. Yi , R. Zhang , J. Spencer , Y. Doi , G. Tian , B. Dong , X. Huang , L.‐F. Yu , D. Gu , H. Ren , X. Chen , L. Lv , D. He , H. Zhou , Z. Liang , J.‐H. Liu , J. Shen , Lancet Infect. Dis. 2016, 16, 161; 26603172 10.1016/S1473-3099(15)00424-7

[26] J. Brem , T. Panduwawala , J. U. Hansen , J. Hewitt , E. Liepins , P. Donets , L. Espina , A. J. M. Farley , K. Shubin , G. G. Campillos , P. Kiuru , S. Shishodia , D. Krahn , R. K. Leśniak , J. Schmidt , K. Calvopiña , M.‐C. Turrientes , M. E. Kavanagh , D. Lubriks , P. Hinchliffe , G. W. Langley , A. F. Aboklaish , A. Eneroth , M. Backlund , A. G. Baran , E. I. Nielsen , M. Speake , J. Kuka , J. Robinson , S. Grinberga , et. al., Nat. Chem. 2022, 14, 15. 10.1038/s41557-021-00831-x 34903857

[27] A. M. King , S. A. Reid‐Yu , W. Wang , D. T. King , G. De Pascale , N. C. Strynadka , T. R. Walsh , B. K. Coombes , G. D. Wright , Nature 2014, 510, 503. 24965651 10.1038/nature13445 PMC4981499

[28] Y.‐H. Yan , G. Li , G.‐B. Li , Medic. Resear. Rev. 2020, 40, 1558. 10.1002/med.21665 32100311

[29] a) W. Gao , L. Zhang , Nat. Rev. Microbiol. 2021, 19, 5; 33024312 10.1038/s41579-020-00469-5 PMC7538279

[30] a) M. C. Sousa , Nature 2019, 576, 389; 31844257 10.1038/d41586-019-03730-x

[31] a) N. Filipczak , J. Pan , S. S. K. Yalamarty , V. P. Torchilin , Adv. Drug Delivery Rev. 2020, 156, 4; 10.1016/j.addr.2020.06.022 32593642

[32] a) T. M. Allen , P. R. Cullis , Adv. Drug Delivery Rev. 2013, 65, 36; 10.1016/j.addr.2012.09.037 23036225

