# Correction to “Liposomal Antibiotic Booster Potentiates Carbapenems for Combating NDMs‐Producing *Escherichia coli*”

**DOI:** 10.1002/advs.202506344

**Published:** 2025-05-08

S. Wu, Y. Wei, Y. Wang, Z. Zhang, D. Liu, S. Qin, J. Shi, J. Shen, Liposomal Antibiotic Booster Potentiates Carbapenems for Combating NDMs‐Producing *Escherichia coli*. Adv. Sci. 2024, 11, 2304397.

https://doi.org/10.1002/advs.202304397

In the Supporting Information of the original publication, the “Lipo@cy5” image of Figure S12b was mistakenly presented, which presented the “Control” image (Figure 4b) during the layout of the images. The corrected figures of Figure S12b are shown below.


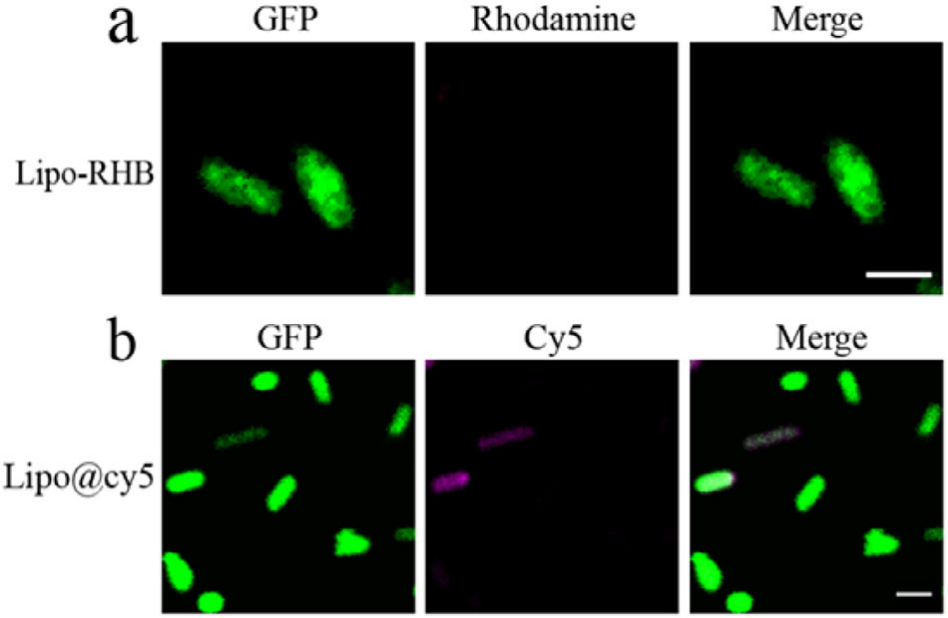


We apologize for this error.

