# Supplementary material for: Liposomal Antibiotic Booster Potentiates Carbapenems for Combating NDMs‐Producing Escherichia c oli
Source: Adv Sci (Weinh). 2023 Nov 7;11(2):2304397. doi: 10.1002/advs.202304397 (PMC10787095; doi:10.1002/advs.202304397)
Supplement: Supplementary file 1 — Supporting Information [file ADVS-11-2304397-s001.pdf]

ADVANCED SCIENCE
Open Access

Supporting Information

for *Adv. Sci.*, DOI 10.1002/advs.202304397

Liposomal Antibiotic Booster Potentiates Carbapenems for Combating NDMs-Producing *Escherichia coli*

Sixuan Wu, Yongbin Wei, Yang Wang, Zhenzhong Zhang, Dejun Liu, Shangshang Qin, Jinjin Shi* and Jianzhong Shen**

Supporting Information

Liposomal antibiotic booster potentiates carbapenems for combating NDMs-producing Escherichia coli

Sixuan Wu[1,2,3,5#], Yongbin Wei[1,2,3#], Yang Wang[6,7#], Zhenzhong Zhang[1,2,3,4], Dejun Liu[6], Shangshang Qin[1,2,3,4*] Jinjin Shi[1,2,3,4*], Jianzhong Shen[6,7*]

[1] School of Pharmaceutical Sciences, Zhengzhou University, Zhengzhou 450001, China

[2] Henan Key Laboratory of Targeting Therapy and Diagnosis for Critical Diseases, Zhengzhou University, Zhengzhou, 450001, China

[3] Key Laboratory of Advanced Drug Preparation Technologies, Ministry of Education, Zhengzhou University, Zhengzhou, 450001, China

[4] State Key Laboratory of Esophageal Cancer Prevention & Treatment, Zhengzhou, 450001, China

[5] School of Life Science, Zhengzhou University, Zhengzhou, 450001, China

[6] Engineering Research Center for Animal Innovative Drugs and Safety Evaluation, Ministry of Education, College of Veterinary Medicine, China Agricultural University, Beijing, China

[7] Beijing Key Laboratory of Detection Technology for Animal-Derived Food Safety, College of Veterinary Medicine, China Agricultural University, Beijing, China

[#]These authors have contributed equally to this work

*Corresponding authors. E-mail addresses: sjz@cau.edu.cn (JS); shijinyxy@zzu.edu.cn (JS); qinshangshang@126.com (SQ).

MATERIALS AND METHODS

Materials. Meropenem (Catalog no. MO108A) was purchased from Meilunbio. Bismuth Potassium Citrate (BPC) Granules was purchased from Livzon Pharmaceutical Group Inc. RNeasy Protect Bacteria Mini Kit (Catalog No. 74524) was purchased from Qiagen. The SDS-PAGE Sample Loading Buffer (Catalog no. P0015F), Reactive Oxygen Species Assay Kit (ROS Assay Kit, Catalog no. S0033S) were purchased from Beyotime Biotechnology. Anti-NDM-1 antibody (Catalog no. NBP1–77688) was purchased from NOVUS Biologicals. The GAPDH Rabbit Polyclonal antibody (Catalog no. 10494-1-AP), and Peroxidase-conjugated Affinipure goat anti-rabbit IgG secondary (Catalog no. SA00001–2) were purchased from Proteintech. The New Delhi metallo-beta-lactamase-1 (NDM-1) ELISA kit (Catalog no. CSB-E14924) was purchased from CUSABIO. CRP ELISA Kit (Catalog no. ml001166), SAA ELISA Kit (Catalog no. ml001976), PCT ELISA Kit (Catalog no. ml002192) Shanghai Enzyme-linked Biotechnology Co. Ltd. Cyanine5 carboxylic acid (Cy5, Catalog no. 2136046) were purchased from J&K Scientific. Maltodextrin (Catalog no. 6363-53-7), Hoechst 33342 (Catalog no. C0030-50 mL), Mueller-Hinton Agar (MHA, Catalog no. M8550-250g), Mueller-Hinton Broth (MHB, Catalog no. M8556-250g), LB broth (Catalog no. L8291-250g), LB agar (Catalog no. L8290-250g), cholesterol (Catalog no. 57-88-5), chloramphenicol (Catalog no. L1311), Kanamycin sulfate (Catalog no. K1030), were purchased from Solarbio. DSPE-PEG2000-Maltodextrin, EggPC were purchased from Ruixi Biotech Co. Ltd. Poly-L-lysine (Catalog no. P856789) was purchased from Macklin. Cell Meter™ Intracellular Fluorimetric Hydrogen Peroxide Assay Kit was purchased from AAT Bioquest (Catalog no.11503)

Characterization: The ultraviolet absorbance and fluorescence values were measured with a microplate reader (Synergy H1, BioTek, USA). Transmission electron microscope (TEM) images were captured by transmission electron microscope (JEM 1200EX, JEOL, Japan). Scanning electron microscopy (SEM) images were obtained by a biological scanning electron microscope (SU8010, Hitachi, Japan). The results of ICP-MS were calculated using an inductively coupled plasma mass spectrometer (7500CE, Agilent, USA). In vivo imaging of bacterial infections with M-MFL was visualized by a small animal imaging system (Xtreme, Bruker, Germany). A High-Performance Liquid Chromatograph (LC02-01, Waters)

was used to monitor the concentration of meropenem in the blood. Blood routine were analyzed by Auto Hematology Analyzer (BC-2800vet, Mindray, China). Blood biochemistry test were conducted by the Auto bio-chemical analyzer (Chemray 240, Rayto, China).

Identification of NDM-1-producing strain: In brief, NDM-1-producing *E. coli* was inoculated in the 2 mL LB medium under shaking 200 rpm at 37°C for 6 h. The *E. coli* was collected by centrifugation at 4000 g for 10 min and washed with PBS. The TE buffer (10 mM Tris Cl, 1 mM EDTA, pH 8.0) containing 1 mg/mL lysozyme was used to lyse bacteria for 5 min. The mixtures were incubated for 5 min at room temperature and centrifuged for 10 min at 5000 g. The residual supernatant was removed by dumping and the 500 μL of TE buffer containing lysozyme was added into the tube. The mixture was vortexed for 10 s and incubate at room temperature for 5 min. During incubation, a vortex for 10 s at least every 2 min is conducted. The 2 mL of Buffer RLT was added into the tube and vortex vigorously. The pellet was centrifuged for 5 min at 5000 g. The 500 μL anhydrous ethanol was added and mix vigorously. The supernatant was transferred to RNasey Spin Column and centrifuged at 12000 rpm. The filtrate was discarded. The 700 μL RW1 was added and centrifuged at 12000 rpm for 15 s. The last operation was repeated. Centrifugation at maximum speed for 1min remove all ethanol. The RNasey Spin Column was placed in a 1.5mL EP tube and added with 30 RNAse-free H_2O for collecting the bacterial mRNA by centrifugation at 12000 rpm for 1 min. The qRT-RCR was measured by Sangon Biotech for confirming the presence of *bla*NDM-1.

Synthesis and characterization of BiNCs: Bismuth potassium citrate (BPC) granule (1 g) was dissolved in 5 mL of ultrapure water. The solution was irradiated under a 300-W UV lamp for 5 min, and then centrifuged at 12000 g for 30 min. The precipitate was washed with ultrapure water and centrifuged again. The bismuth nanoclusters (BiNCs) is collected and stored at 4 °C away from light.

Checkerboard assay: Briefly, a serial 2-fold diluted solution of 25 μL of meropenem and BiNCs were added into each well of a 96-well plate[(48)]. Then 50 μL of freshly prepared bacterial suspension (~10^5CFU/mL) in MHB was added to the well. The plates were then incubated at 37 °C with shaking for 16h-18 h [(49)]. Finally, the OD_{600} of the plates was

measured by microplate reader. The MICs were defined as the lowest concentrations of antibiotics with no visible growth of bacteria.

Time-Kill Assay: A single colony of NDM-1 *E. coli* 1322 was inoculated in 2 mL of MHB overnight at 37 °C with shaking of 200 rpm. The bacterial density was diluted 10,000 times and then cultured for another 2 h. After incubating, the bacterial suspension was treated with MEM+BiNCs+H_2O_2 MEM+BiNCs, MEM, BiNCs, and H_2O_2. MHB with no drugs served as a control. Aliquots of each culture were collected at different time intervals (0, 0.5, 1, 2, 4, and 6 h). The aliquots were 10-fold serially diluted in PBS, then plated onto agar plates. After incubation at 37 °C, bacterial single colonies were calculated from the colonies growing on the plates after overnight culture.

Zn(II) displacement analysis by ICP-MS: First, different concentrations of BiNCs (0, 50 μg/mL, 100 μg/mL, 200 μg/mL, 50 μg/mL, 400 μg/mL, 600 μg/mL) were treated with hydrogen peroxide solution (100 μM) at 37°C overnight. Then the purified NDM-1 protein was diluted 500 times with PBS. Purified NDM-1 diluent (200 μg/mL) was incubated with different concentrations of BiNCs at 25°C for 2 h with mild shaking. The mixed solution was centrifuged to remove BiNCs at 12000 rpm for 30 min, and the unbound Bi(III) and Zn(II) were removed by dialysis. Dialysate and raw solution were subsequently analyzed using an inductively coupled plasma mass spectrometer (7500CE, Agilent, USA).

IC50 enzyme inhibition assay: Various concentrations of BiNCs were incubated with 100 μM hydrogen peroxide for 12 h at 37 °C. After incubation, the BiNCs were centrifuged at 12000 rpm for 30 min. The supernatants were mixed with purified NDM-1 (4μg/mL) for 1 h at 25 °C in a 1:1 ratio. Then 100 μL mixture was transferred to a 96-well plate and added 1.2 mM meropenem into each well. The OD_{300} was monitored every 2 min for 30 min by a UV-Vis spectrometer (UV2700, Shimadzu, Japan).

In Vitro Hydrogen Peroxide-Triggered Bi(III) Release: The solutions of BiNCs (200μg/mL) were incubated with different concentrations of hydrogen peroxide(0, 20 μM, 50 μM, 100 μM, 200 μM) at 37°C for 12h. Then, the solutions of BiNCs were centrifuged to obtain the precipitates and supernatants. The precipitations were applied to dynamic light scattering (DLS). The contents of Bi(III) were detected in the supernatants by ICP-MS.

Cellular thermal shift assay. The NDM-1 engineering bacteria *E. coli* (NDM-star) was cultured overnight at 37°C in LB medium (including Kanamycin sulfate 35 μg/mL). The bacterial suspension was diluted twice and the Isopropyl-beta-D-thiogalactoside (IPTG) was added to the suspension to reach the concentration of 50μM. The mixture was cultured at 37°C for 1 h and 20°C for 2 h. After induction of NDM-1 expression, the BiNCs and H_2O_2+BiNCs were respectively added into the bacterial suspension and incubated at 20°C for 3 h. The control experiment was also performed in the absence of BiNCs and H_2O_2+BiNCs under the same conditions. The bacterial pellets were harvested by centrifugation and washed with PBS three times. The obtained bacterial pellets were re-suspended in the PCR tube and heated for 3 min in a 96-well thermal circulator at the specified temperature. The sample was immediately cooled at 25□ and subjected to 3 cycles of heat treatment. To obtain the total protein, the cells were collected, washed, and the total proteins were extracted by boiling in the lysis buffer including PBS and 1 mM PMSF for 10 min. NDM-1 protein bands were detected by Western blotting.

Michaelis–Menten kinetics BiNCs was dissolved in hydrogen peroxide solution into different concentrations (0, 25 μg/mL, 50 μg/mL, 100 μg/mL, 200 μg/mL) and subsequently incubated at 37 °C overnight. Purified NDM-1 (200 μg/mL) was incubated with BiNCs at 37°C for 1h with mild shaking. The mixture was centrifuged to remove BiNCs at 12000 rpm for 30 min, then 150 μL supernatant was mixed with 50 μL MEM in a 96-well microplate, resulting in the concentration of MEM ranging from 25 μM to 1.6 mM. Finally, the absorbance of each well in the 96-well plate at 300 nm was monitored every 2 min for 30 min. The bacterial pellets were harvested and washed with PBS three times. The 400 μL of PBS containing PMSF (1 mM) was added to bacterial pellets. The bacterial suspensions were aliquoted into PCR tubes and heat treatment was performed at the designated program which is the temperature ranging from 38.4 °C to 82.5 °C for 3 min and then down to 25 °C for three cycles in a PCR instrument. For the cell lysis, the samples were first fully mixed with 6 × SDS PAGE buffer and vortexed for 3 min. The samples were added to boiling water to treat for 10 min and centrifuged to obtain the supernatant. All the samples were subjected to western blot analysis for detection and quantification of NDM-1 content as described above.

Synthesis and Characterization of Maltodextrin-Modified Lipo (M-MFL): M-MFL was prepared by using a standard vesicle extrusion method. Briefly, EggPC, cholesterol, and DSPE-PEG-MA were dissolved in 4 mL chloroform and mixed at a 6:1:3 weight ratio (16 mg total weight) in around bottom flask. The chloroform was evaporated under reduced pressure to form a thin lipid layer. The resulting lipid film was rehydrated by adding 2 mL PBS, followed by 5 min of bath sonication to produce multilamellar vesicles. Finally, a Ti-probe was used to sonicate the multilamellar vesicles for 2 min at 100 W to produce homogeneous opalescent dispersions. The final liposomes were stored in tight containers at 4 °C in the dark until use. Then non-encapsulated drug, molecules were removed by dialysis using a 100KD dialysis membrane. The final liposomes were stored in tight containers at 4 °C. The common liposomes ((DOPC/DOPE/Chol; 2:1:1, molar ratio) were prepared according to the above mentioned thin film hydration method.

The hydrodynamic size (diameter, nm) and ζ potential (mV) of M-MFL and bare liposomes were measured using DLS. The morphology of liposomes was observed under transmission electron microscopy (TEM) and cryoelectronic microscopy (cryo-EM). The bismuth element encapsulated into liposomes was detected by transmission electron microscopy mapping.

ROS Measurement: Bacterial production of reactive oxygen species was detected by a ROS fluorescence probe of 2,7-dichlorodihydrouorescein diacetate (DCFH-DA). *E. coli* (1322) was cultured overnight in MHB at 37 ºC. The bacteria were collected and suspended in 10 μM DCFH-DA with a bacterial concentration of 10^6 to10^7 CFU, and incubated in a cell incubator at 37 □ for 30 min. The bacteria were washed with PBS three times to fully remove the non-intracellular DCFH-DA. 1 mL MFL (1 mg/mL) suspension was mixed with 1×10^7 CFU *E. coli*. After 30 min incubation, the bacteria were collected by centrifugation at 4,000×g for 10 min, and suspended in PBS. Samples were examined by flow cytometer and laser confocal microscope.

Intracellular Hydrogen Peroxide Content Measurement: Bacterial hydrogen peroxide content was determined by intracellular fluorescence hydrogen peroxide detection kit (Catalog no. 11503). A single colony of NDM-1 *E. coli* (EC 1322) was inoculated in the 2

ml MHB medium under shaking at 200 rpm at 37 °C for 12 h. The bacterial suspension was diluted 10 times into four tubes. Then, stain bacteria with Green Peroxide Sensor working solution and incubate for 30min. the *E. coli* was collected by centrifugation at 4000 g for 10 min and washed with PBS. Finally, the different concentration M-MFL was added into each tube containing *E. coli* suspension at 37 ºC for 30 min. After incubation, the *E. coli* suspension was centrifuged at 4000 g for 10 min and washed with PBS. The fluorescence intensity was monitored at Ex/Em = 488/525 nm.

H_2O_2-triggered drugs release: MB and M-MFL@MB were dispersed in 1 mL of PBS with different concentrations H_2O_2 (1,10,100,200μM), placed in dialysis bags (molecular weight cut-off = 100 KDa), and then dialyzed against solutions with the same H_2O_2 concentration. The concentration of meropenem in dialysate was measured at various time intervals by microplate reader (OD_{300}). The release rate of meropenem was calculated by the equation: [total mass of released meropenem/ amount of loaded meropenem] × 100%.

Bacteria adherence assay: A single colony of NDM-1 *E. coli* (EC1322) was inoculated in the 2 ml MHB under shaking at 200 rpm at 37 °C for 12 h. 1 mL culture was centrifugated at 4000 g for 5 min and washed with PBS. Firstly, the bacteria were fixed with 4% paraformaldehyde for 1 h at 4 °C. The fixed bacteria were suspended in M-MFL@B, MFL@B (1mg/ml), and PBS to a concentration of 1×10^8 CFU/ml at 37 °C for 30 min. Subsequently, bacteria were centrifugated at 4000 g and washed with PBS twice for removing unbound nanoparticles. Finally, The purified bacteria were observed by Scanning electron microscopy (SEM).

Bacteria selective targeting assays: Poly-L-lysine was diluted 10 times for bacterial adhesion. The 1×poly-L-lysine was added to cover glass placed in a 6-well plate at 37°C for 1 h. The 6-well plate was sent to cell incubator to dry. After 12 h, RAW264.7 cell suspension (1.5×10^5 per well) was seed in a poly-L-lysine-binding 6-well plate and allowed to adhere for 12 h. After removing the culture medium, the Hoechst 33342 (10 μg/ml) was added to the 6-well plate for 20 min, PBS was used to wash the 6-well plate to get rid of free probe. The 100μl E. coil expressing green fluorescent protein (GFP) was dispersed into a 6-well plate for adherence. After binding to the cover glass, the 6-well plate was treated with M-MFL and

MFL for 30 min, and PBS as a control then washed with PBS. Finally, the cover glass was preserved and used for confocal laser microscopy imaging.

M-MFL-Bacteria Fusion Studies: DMPE-Rhb (0.5 mol %) was mixed with EggPC, DSPE-PEG-MA and cholesterol. The synthesis of membrane fusion liposome was constructed according to the above protocol of maltodextrin-modified Lipo (M-MFL). And then was subsequently incorporated into a bilayer of liposomes to form a fluorescent liposome (1 mg/mL). For CLSM assay: The resulting M-MFL solutions (50 μg/mL) were mixed with 1×10^7 CFU GFP-expressing *E.coli* at 37 ºC for 20 min. After incubation, samples were centrifuged at 4000 g for 10 min. The bacteria were washed and resuspended in 1 mL PBS. The fluorescence image was then obtained by Confocal Laser Scanning Microscopy. Flow cytometry was also used to evaluate the membrane fusion ability of M-MFL. For bio-TEM assay, the above obtained M-MFL or M-MFL@MB (50 μg/mL) was incubated with 1×10^7 CFU/mL bacteria. After incubation for 8 h, the bacteria was obtained at 4000 g centrifugation for 10 min. The bacteria sample was sent to the company of science compass for bio-TEM detection.

In vitro antibacterial activity assay. 50 μL bacterial suspension containing 1×10^5 CFU/ml *E. coli* 1322 was mixed with 50 μL various concentrations of M-MFL@MB in 96-well plate. After incubation for 16 h, 10 μL of MTT solution (5 mg mL^{-1}) was added to 96-well plate, then incubated for 4 h. Subsequently, 100 μL of DMSO was added to the 96-well plate to dissolve the Formazan. Finally, the A_{570} was measured to indirectly reflect the number of living bacteria.

Isolation of *E. coli* Outer Membrane Vesicles (OMVs): A clinical isolate expressing NDM-1 *E. coli* (NDM-1 EC1429) Outer Membrane Vesicles (OMVs) was collected according to published protocols. A single colony of NDM-1 *E. coli* (NDM-1 EC1429) was inoculated in the 10 ml MHB medium under shaking at 200 rpm at 37 °C for 12 h. The obtained *E. coli* suspension (2 mL) was diluted 100 times in 200 ml MHB and cultured in 1 L Erlenmeyer flask under shaking at 200 rpm at 37 °C for 4 h. Then, the Lipo, M-MFL (9 mg/ mL) were separately added into *E. coli* suspension (200 mL) and cultured at 37 °C when the OD_{600} of the culture was = 1.0. The *E. coli* suspension was centrifuged at 12000 rpm for 30

min to remove the bacteria in MHB. To remove bacteria completely, the medium was filtered with a 0.22 μm vacuum filter twice. The supernatant obtained by centrifugation was ultracentrifuged at 1700 000 × g for 2 h at 4 °C. The outer membrane vesicles (OMVs) were resuspended in 1mL PBS and stored at −80 °C.

In vivo bacteria targeting assays: Briefly, the 72 hpi zebrafish were soaked in the 10^6 CFU recombinant *E coli* LB21 expressing green fluorescent protein (GFP) solution for 3h. Then, the zebrafish were transferred to fresh medium to wash off the unbound bacteria three times. Subsequently, M-MFL (1 mg/ mL) labeling Rhb and Lipo labeling Rhb were added into six-well cell culture plates containing fresh medium and zebrafish for 0.5 h, respectively. Then, the zebrafish were washed with fresh medium three times for CLSM imaging.

Female BALB/c mice (female, 19–21g) were anesthetized with 10 mg/mL pentobarbital sodium by intraperitoneal injection at 120 μL. A suspension of 10^8 CFU *E. coli* (EC.1322) in 100 μL saline was injected into the right leg, and 100μL of saline containing LPS (0.2mg/ml) was injected into left leg. After 1 h, the mice were respectively injected with M-MFL@IR783, MFL@IR783, and IR783 at 100μg/ml IR783 equivalent dose of by tail intravenous. Fluorescence images were captured in both legs at 0 h,1 h,2 h,4 h, 6 h, 8 h, 20 h, and 22 h after the injection of M-MFL@IR783, MFL@IR783, and IR783. Mice were euthanized, and the bacterial-infected and saline-treated muscles were collected and analyzed by histology for the presence of bacteria.

Animal tests: BALB/c mice (female, 6–8 weeks, ≈19–21g) were housed in SPF condition. All animal rooms had a controlled environment with a temperature of 25 °C and 55% relative humidity. The zebrafish were obtained from the Shanghai FishBio Co., Ltd (China). All animal studies were carried out following the guidelines of the Regional Ethics Committee for Animal Experiments and the Care Regulations approved by the Institutional Animal Care and Use Committee of Zhengzhou University. The license number of the experimental animal is No.410975211100031648.

Mouse pneumonia model: The mice were anesthetized by intraperitoneal injection of pentobarbital sodium 10 mg/mL at a dose of 60 mg/kg. The mouse pneumonia model was successfully established with 1×10^8 CFU *E. coli* (NDM-EC1322) via a nasal drip. After

infection for 1 h, the pneumonia model mice were treated with MEM, M-MFL (1.8125 mg/mL), BiNCs, MB, M-MFL_{MEM}, M-MFL@MB at 10 mg/kg meropenem equivalent dose by intraperitoneal injection. We then gave an additional treatment every six hours. After 24 h post-infection, mice were sacrificed for collecting lung tissues and blood. Afterward, the lung tissues were homogenized for colony counting. 200 μL of the blood was used in blood routine analysis. The inflammatory indicators (Procalcitonin PCT, C-reactive protein CRP, and serum amyloid A1 protein, SAA1) of bacterial infection were detected by Elisa kit.

Mouse sepsis model: Bacterial suspension (4×10^6 CFU mL^{-1}, 100μL) was injected into mouse abdominal cavity to establish a sepsis model. After infection for 1 h, the pneumonia model mice were treated with MEM, M-MFL, BiNCs, MB, M-MFL@MEM, M-MFL@MB at 10 mg/kg meropenem equivalent dose by intraperitoneal injection. We then gave an additional treatment every six hours. After 24 h post-infection, mice were sacrificed for collecting hearts, spleen, kidney tissues, and blood for bacteria counting. The hematoxylin and eosin (H&E) staining was utilized to observe the morphologies of tissues. The inflammatory indicators (Procalcitonin PCT, C-reactive protein CRP, and serum amyloid A1 protein, SAA1) of bacterial infection were detected by the corresponding Elisa kit.

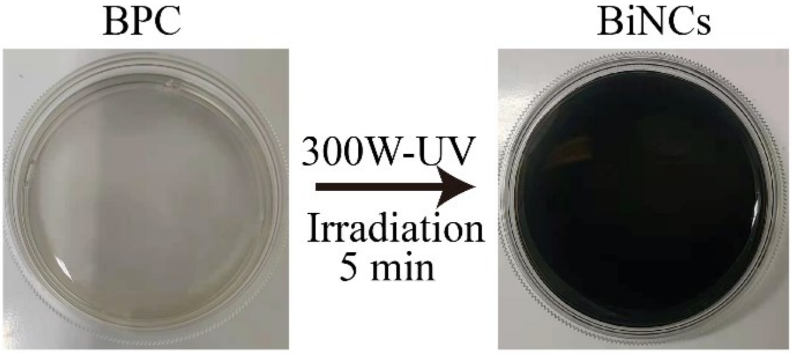

Fig. S1 Representative digital photograph of BPC (left) and BiNCs (right). BPC is converted to BiNCs via one UV irradiation, accompanied by a change in color of the solution from colorless to black.

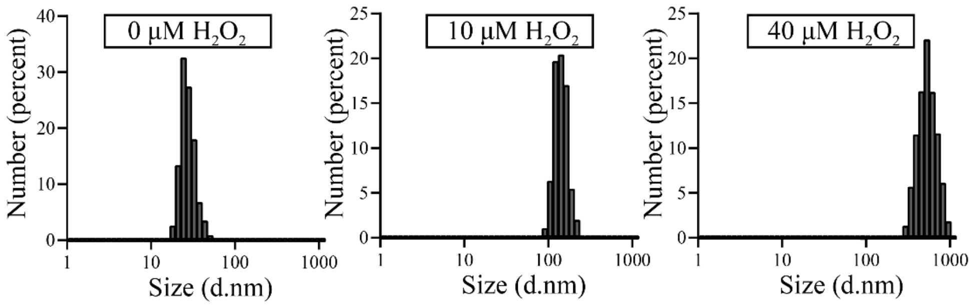

Fig. S2 Representative size distribution of BiNCs incubated in PBS containing different concentrations of H_2O_2 (0, 10 and 40 µM). Results showed that BiNCs undergo morphological changes under PBS containing 10 µM and 40 µM H_2O_2, respectively, while maintaining the structural integrity in PBS alone, reflecting that BiNCs is highly sensitive to ROS.

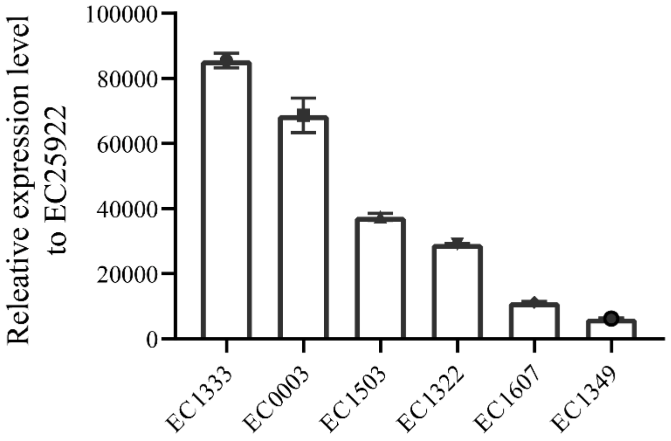

Fig. S3 Verification of blaNDM gene expression. The *bla*NDM-1 was determined in six clinical *E.coli* isolates, and *E.coli* ATCC25922 was used as negative controls. The six clinical isolates all express *bla*NDM-1 gene, suggesting that they are genetically carbapenems resistant. Data are presented as mean values ± SD, n=3 biologically independent samples.

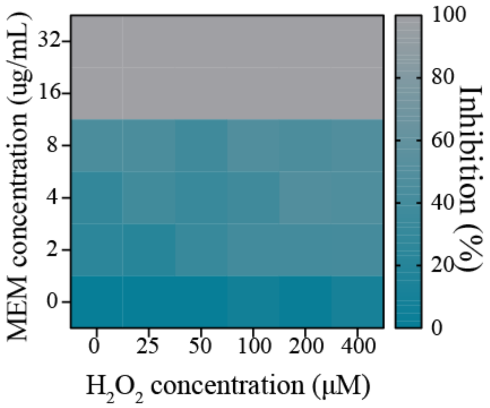

Fig. S4 Representative heat plots of microdilution checkerboard assays for the combination of H_2O_2 and meropenem against NDM-1 EC1322. Results demonstrated that there is no synergistic interaction between H_2O_2 with MEM toward NDM-1 EC1322.

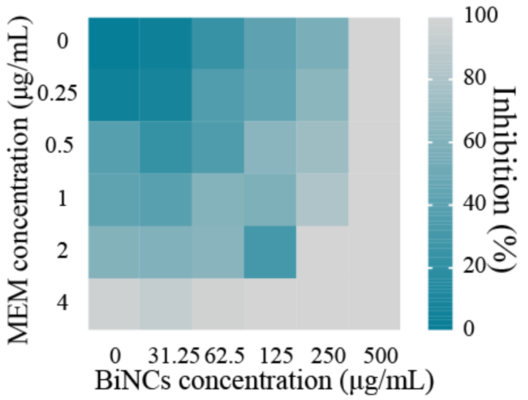

Fig. S5 Representative heat plots of microdilution checkerboard assays for the combination of BiNCs and meropenem in the presence of H2O2 against NDM-1 negative *E.coli*. The result reflected that BiNCs could not reduce MIC values of MEM toward NDM-1 negative strain in the presence of H_2O_2.

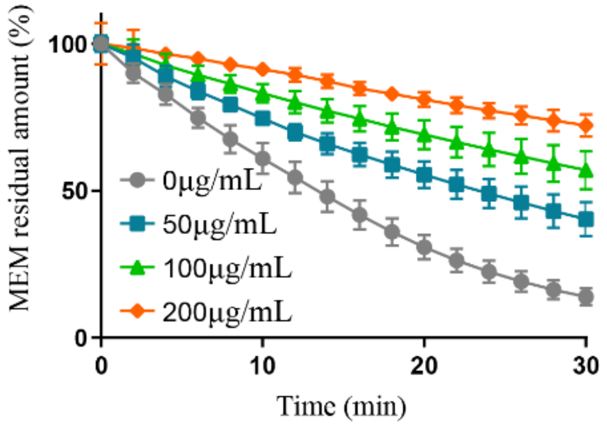

Fig. S6 Hydrolytic effects of NDM-producing *E.coli* BL21 after treated with different concentrations of BiNCs in the presence of 200 μM H_2O_2 on meropenem (n=3). The result showed that the activity of NDM-1 decreased as the 200μM H_2O_2-pretreated BiNCs concentration escalated, ultimately leading to the activities of NDM-1 being inhibited by approximately 80%. Data are presented as mean values ± SD, n=3 biologically independent samples.

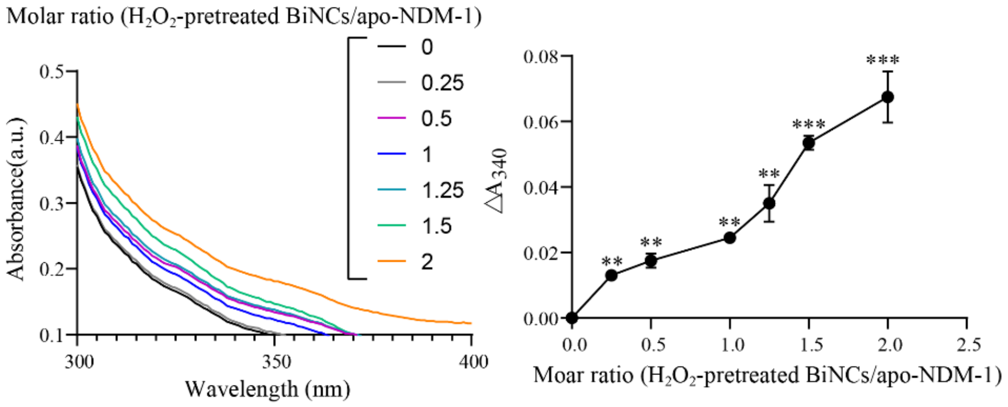

Fig. S7 Different UV-vis spectra of apo-NDM-1 upon addition of different concentrations of H_2O_2-pretreated BiNCs. The right presents the changes of absorbance at 340 nm. The appearance of an absorption band at 340 nm after incubating apo-NDM-1 with the supernatant (containing Bi(III)) from 200μM H_2O_2-pretreated BiNCs was observed, which is characteristic for Bi–S ligand-to-metal charge transfer (LMCT) band, suggesting that the Bi(III) ions released from BiNCs could bound to NDM-1. Data are presented as mean values ± SD, n=3 biologically independent samples. Statistical significance was analyzed by the two-tailed Student's t-test. $^*P < 0.05$, $^{**}P < 0.01$, $^{***}P < 0.001$, $^{****}P < 0.0001$.

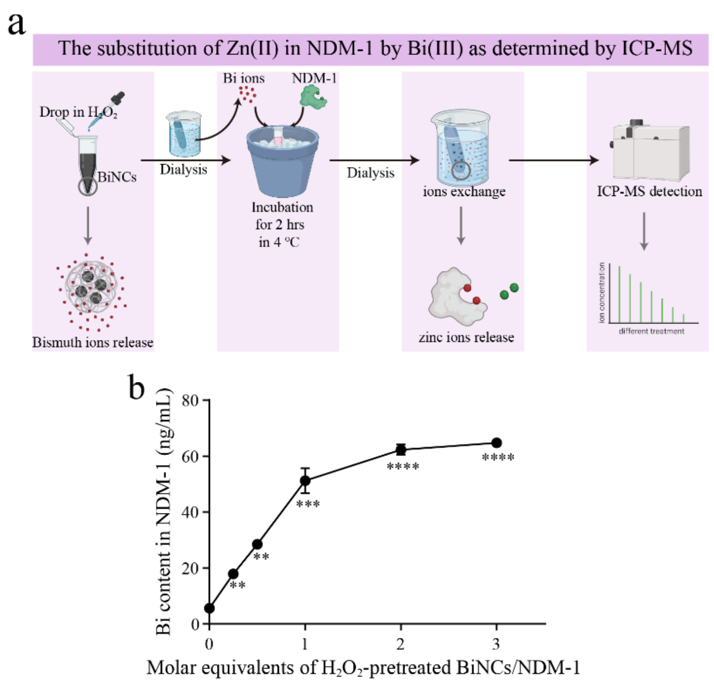

Fig. S8 The substitution of Zn(II) in NDM-1 by H_2O_2-pretreated BiNCs as determined by ICP-MS. a, b, The experimental scheme (a) and result (b) of Bi(III) contents in NDM-1 after H_2O_2-pretreated BiNCs treatment. The result presented that the content of Bi(III) in NDM-1 exerted a significant increase with the addition of H_2O_2-pretreated BiNCs, reflecting the direct binding of Bi(III) in NDM-1 induced by H_2O_2-pretreated BiNCs. Data are presented as mean values ± SD, n=3 biologically independent samples. Statistical significance was analyzed by the two-tailed Student's t-test. *$P < 0.05$, **$P < 0.01$, ***$P < 0.001$, ****$P < 0.0001$.

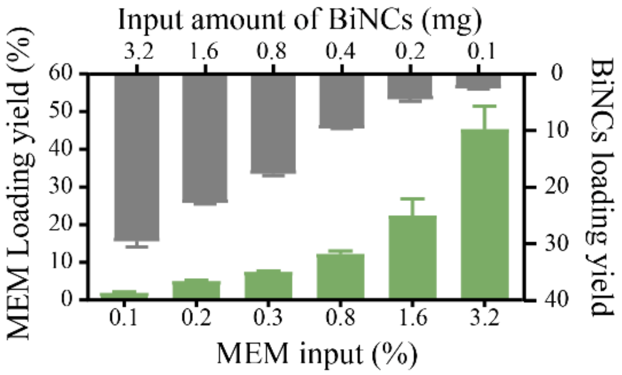

Fig. S9 Loading yield at various meropenem (MEM) and BiNCs inputs (n=3). It can be seen that a 50% loading yield of MEM into BiNCs was obtained by using an optimal input of 3.2 wt%. Then, the MB loading efficiency in M-MFL@MB was calculated to 29.5% with an input of 3.2 wt%. Data are presented as mean values ± SD, n=3 biologically independent samples.

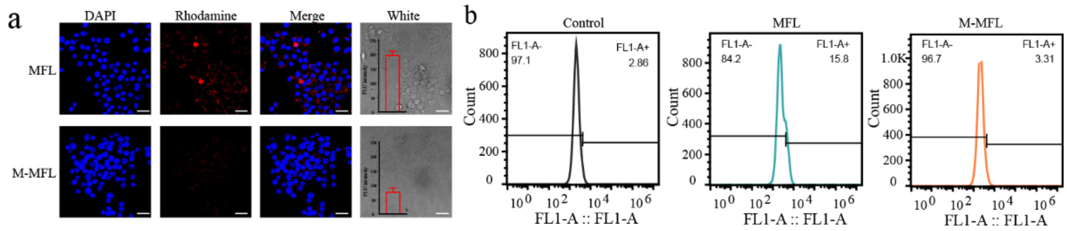

Fig. S10 Immunocompatibility analysis. a, b, Evaluation of Immune escape ability of rhodamine-labeled MFL and M-MFL via CLSM (a, Scale bar, 10 μm) and flow cytometer (b) assays, respectively. Untreated mononuclear macrophage (RAW264.7) was used as control. The embedded picture (a) shows semi-quantitative analysis of red fluorescence. Benefiting from the specific maltodextrin transporter of bacteria, M-MFL presented a more effective immune escape ability compared with MFL. Data are presented as mean values ± SD, n=3 biologically independent samples. Statistical significance was analyzed by the two-tailed Student's t-test. *$P < 0.05$, **$P < 0.01$, ***$P < 0.001$, ****$P < 0.0001$.

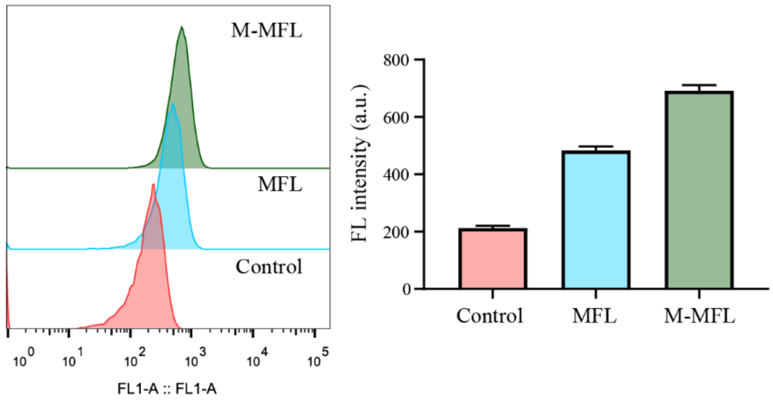

Fig. S11 Flow cytometry analysis of membrane fusion efficacy. Flow cytometry assay and the corresponding fluorescence semi-quantitative analysis detect the membrane fusion interaction between M-MFL or MFL with *E.coli*. M-MFL and MFL were labeled with fluorescent dye Rhodamine (red). Control group was incubated with PBS. Results showed that M-MFL-mediated high efficiency of membrane fusion. Data are presented as mean values ± SD, n=3 biologically independent samples.

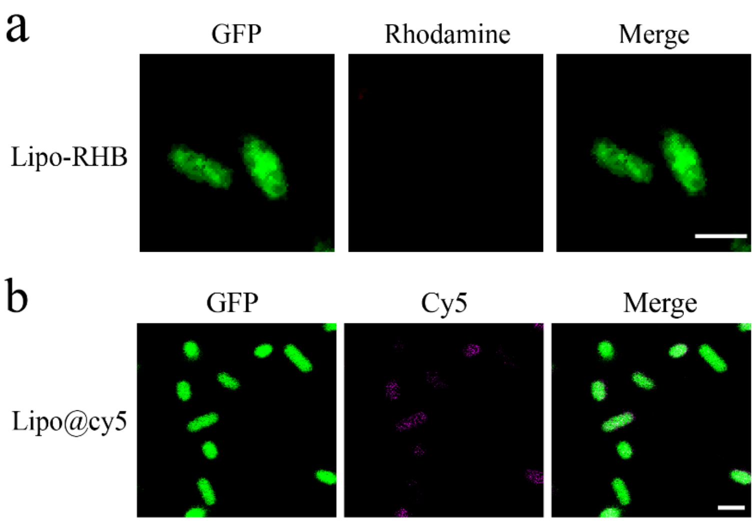

Fig. S12 Membrane fusion and intracellular drug delivery analysis of common liposome. a, b, Representative confocal images visualize the membrane fusion interaction between lipo with *E.coli* (a), and intracellular drug delivery of lipo into *E.coli* (b). Lipo was labeled with fluorescent dye Rhodamine (red), Cy5 fluorescent dye (pink) was loaded into lipo for substituting MB, and the *E.coli* could express GFP (green fluorescent protein, green). Scale bar, 1 μm. The common liposome constituted by soybean lecithin and cholesterol had a negligible membrane fusion ability, thus resulting in the weak efficiency of intracellular delivery.

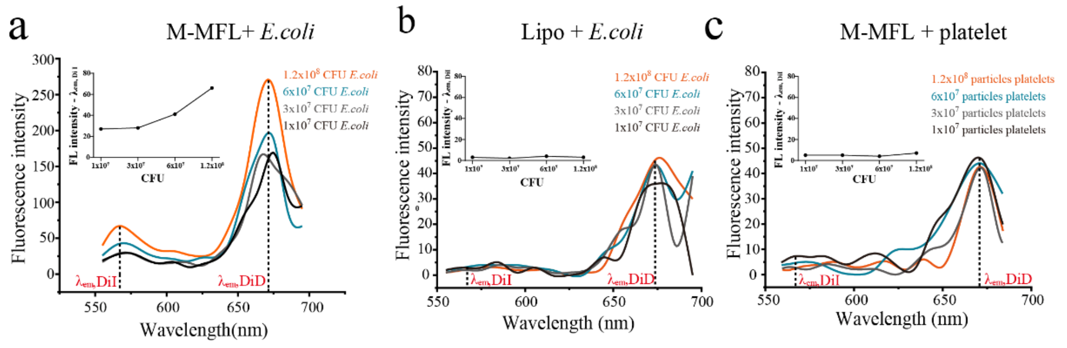

Fig. S13 FRET measurements of the membrane fusion ability. A fluorescent donor (DiI) and a fluorescent acceptor (DiD) were concurrently incorporated into the liposomal nanoparticles so that the acceptor completely quenched the fluorescence emission from the donor. The FRET pair-labeled M-MFL was incubated with different concentrations of *E.coli* or platelet, and FRET pair-labeled lipo was incubated with different concentrations of *E.coli*. Then, the emission intensity of DiI (donor) at 565nm was monitored with the increase of bacterial or platelet concentrations. As observed in Fig. S13, with the amounts of *E.coli* increasing, there was an increase of fluorescence at 565 nm in M-MFL group, suggesting the weakening of FRET interaction due to the successful fusion between M-MFL with *E.coli*. Unsurprisingly, the FRET pair of liposomes after incubating with *E.coli* still maintain a solid FRET effect. Besides, M-MFL hardly fused with platelets, benefiting from MA-mediated selective targeting over bacteria, not mammalian cells.

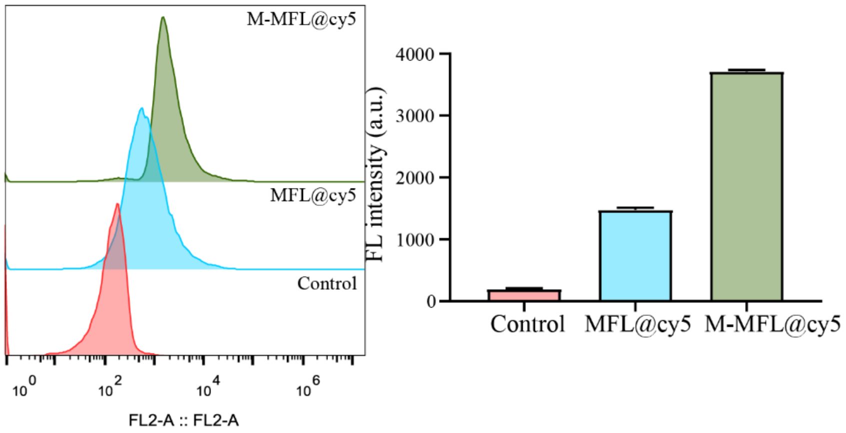

Fig. S14 Flow cytometry analysis of intracellular drug delivery efficacy. Flow cytometry assay and the corresponding fluorescence semi-quantitative analysis (n=3) determine the intracellular drug delivery efficacy of M-MFL or MFL. Cy5 fluorescent dye (pink) was loaded into M-MFL and MFL for substituting MB. Control group was incubated with PBS. Results showed that M-MFL-mediated high efficiency of intracellular drug delivery. Data are presented as mean values ± SD, n=3 biologically independent samples.

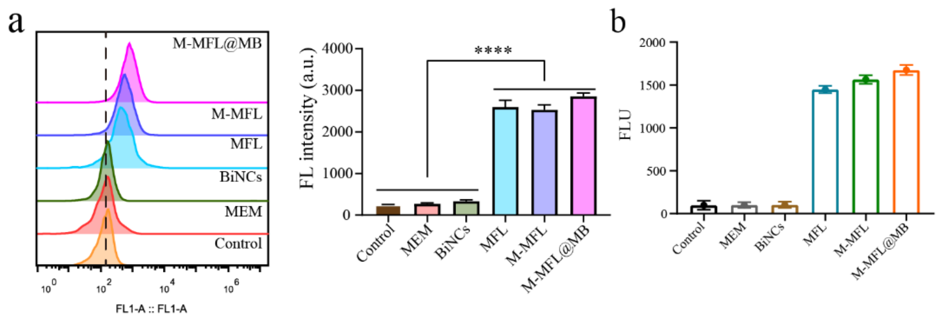

Fig. S15 Flow cytometry analysis and CLSM semi-quantitative analysis of intracellular ROS production. a, Flow cytometry assay and the corresponding fluorescence semi-quantitative analysis (n=3) monitor intracellular ROS levels after being treated with different nanoparticles by detection of DCFH-DA fluorescence intensity. b, CLSM semi-quantitative analysis of Fig. 4f for determining intracellular total ROS level. MFL-mediated membrane fusion strategies could endogenously trigger intracellular ROS production. Data are presented as mean values ± SD, n=3 biologically independent samples. Statistical significance was analyzed by the two-tailed Student's t-test. **P*<0.05, ***P*<0.01, ****P*<0.001, *****P*< 0.0001.

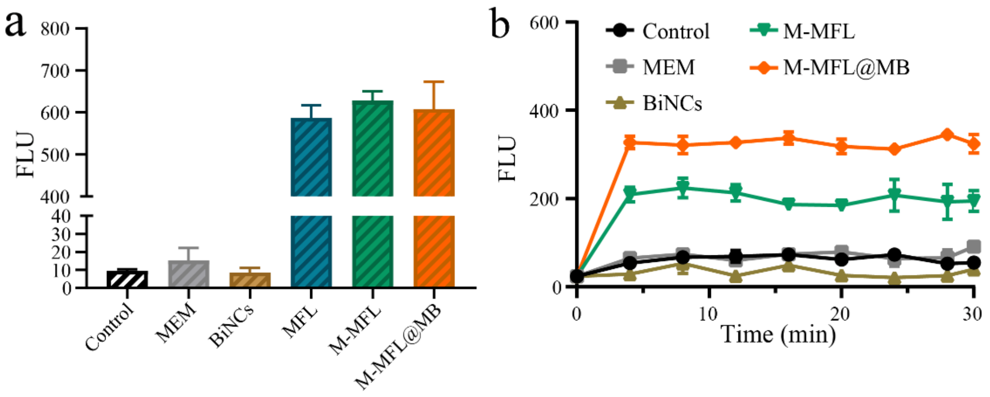

Fig. S16 Intracellular ROS monitor using microplate reader. a, b, Total intracellular ROS level (a) and the real-time kinetic change of intracellular ROS level (b) after being treated with different nanoparticles was monitored by detection of DCFH-DA fluorescence intensity using microplate reader (n=3). Firstly, MFL-mediated membrane fusion strategies could endogenously trigger intracellular ROS production (Fig. S16a). Besides, we found that, after being treated with nanoparticles for 4 min, these groups of MFL, M-MFL and M-MFL@MB reached the highest peak of intracellular total ROS level, which was much higher than other groups (Fig. S16b). And even at 30min, the ROS level in M-MFL and M-MFL@MB groups still existed steadily with no significant decreasing trend (Fig. S16b). These results indicated that membrane fusion strategy initiated a rapid and stable ROS burst inside bacteria. Data are presented as mean values ± SD, n=3 biologically independent samples.

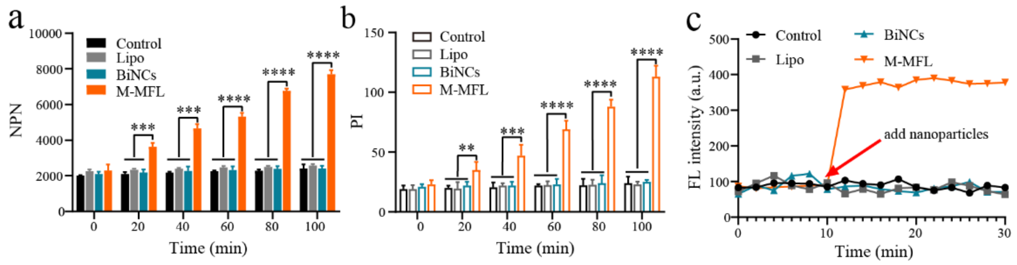

Fig. S17 Detection of membrane permeability and potential. a, b, Outer membrane (OM, a) and inner membrane (IM, b) permeabilization of different nanoparticles treatment was monitored by detecting the fluorescence intensity of NPN (n=3). c, Detection of membrane depolarization after different nanoparticles treatment via DiSC3 (n=3), a membrane potential probe that gathers in the phospholipid bilayer, causing dye self-quenching. When the membrane is depolarized, the potential is lost and the DiSC3 is released into the solution, triggering fluorescence enhancement. we found that M-MFL-mediated membrane fusion strategy presented a strengthened permeability of OM and IM in *E.coli* due to the impaired integrity of bacterial membrane (Fig. S17a and b), which then results in membrane potential depolarization (Fig. S17c). Data are presented as mean values ± SD, n=3 biologically independent samples.

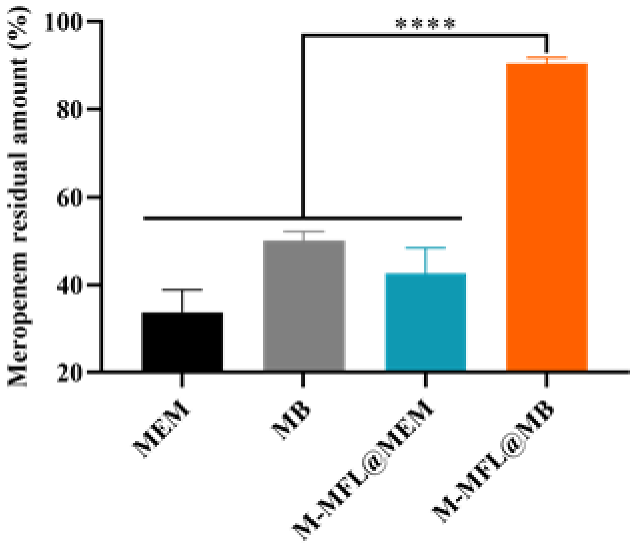

Fig. S18 Hydrolytic effects of different nanoparticles-treated NDM-1-producing E. coli BL21 on meropenem. Measurement meropenem residual amount on NDM-1-producing EC1322 after treated with sub-lethal concentrations of MEM, MB, M-MFL@MEM and M-MFL@MB, respectively. Result showed that more amounts of MEM was retained in M-MFL@MB (~94 %) group than that in MEM (~33 %), MB(~50 %) and M-MFL@MEM (~42 %) groups. Data are presented as mean values ± SD, n=3 biologically independent samples. Statistical significance was analyzed by the two-tailed Student's t-test. *P<0.05, **P<0.01, ***P<0.001, ****P<0.0001.

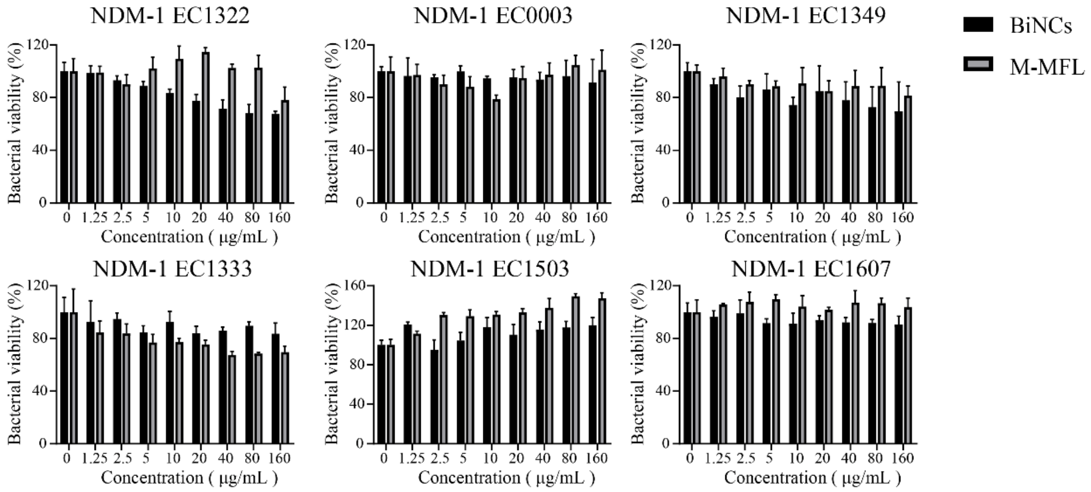

Fig. S19 Assessment of antibacterial ability of BiNCs and M-MFL in vitro. Measurement of bacterial colony-forming units, obtained from six clinical isolates of NDM-1-producing *E.coli* treated with different concentrations of BiNCs and M-MFL, respectively. There is no evident bactericidal activity in BiNCs and M-MFL groups. Data are presented as mean values ± SD, n=6 biologically independent samples.

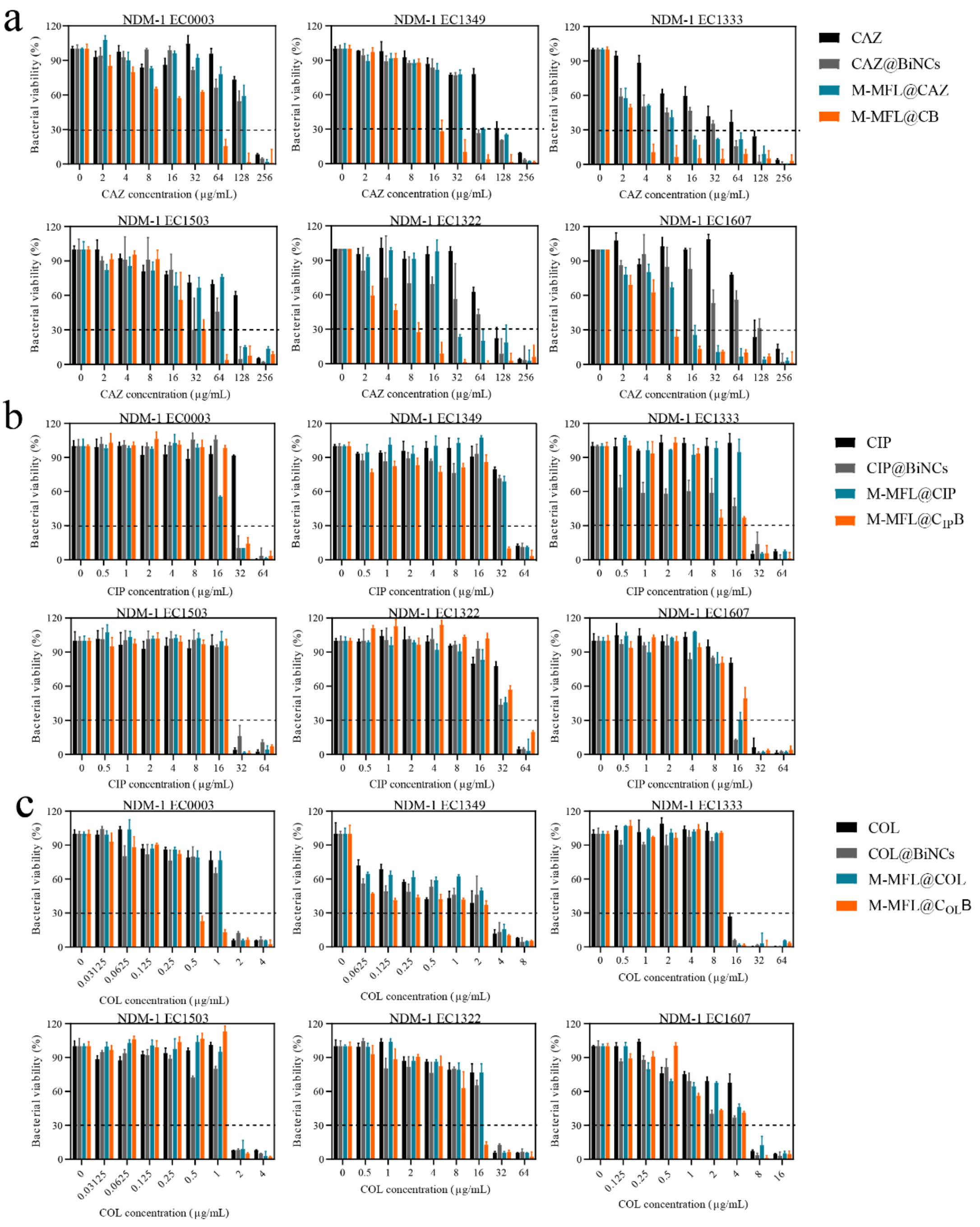

Fig. S20 Bacterial viability of different formulations in vitro. a, Measurement of bacterial colony-forming units, obtained from six clinical isolates of NDM-1-producing *E.coli* treated with different concentrations of ceftazidime (CAZ), CAZ@BiNCs, M-MFL@CAZ and M-MFL@CB, respectively (n=6). b, Measurement of bacterial colony-forming units, obtained from identical *E.coli* treated with different concentrations of ciprofloxacin (CIP), CIP@BiNCs, M-MFL@CIP and M-MFL@C_{IP}B, respectively (n=6). c, Measurement of bacterial colony-forming units, obtained from identical *E.coli* treated with different

concentrations of colistin (COL), COL@BiNCs, M-MFL@COL and M-MFL@C$_{OL}$B, respectively (n=6). We found that the antibiotic booster also showed synergies with CAZ against NDM-1-producing E. coli isolates, but not with CIP and COL. Data are presented as mean values ± SD, n=6 biologically independent samples.

a

MICs in six NDM-1-producing strains (μg/mL).

Strains	CAZ	CAZ@BiNCs	M-MFL@CAZ	M-MFL@CB
NDM-1 EC0003	128	64	64	8
NDM-1 EC1349	128	64	32	4
NDM-1 EC1333	256	128	128	32
NDM-1 EC1503	128	128	32	8
NDM-1 EC1322	256	128	128	32
NDM-1 EC1607	128	128	16	8

b

MICs in six NDM-1-producing strains (μg/mL).

Strains	CIP	CIP@BiNCs	M-MFL@CIP	M-MFL@C$_{IP}$B
NDM-1 EC0003	64	32	32	32
NDM-1 EC1349	64	64	64	32
NDM-1 EC1333	32	32	32	32
NDM-1 EC1503	64	64	64	64
NDM-1 EC1322	64	32	32	32
NDM-1 EC1607	32	16	16	16

c

MICs in six NDM-1-producing strains (μg/mL).

Strains	COL	COL@BiNCs	M-MFL@COL	M-MFL@C$_{OL}$B
NDM-1 EC0003	4	4	4	4
NDM-1 EC1349	2	1	1	1
NDM-1 EC1333	2	2	2	2
NDM-1 EC1503	2	2	2	1
NDM-1 EC1322	4	4	2	2
NDM-1 EC1607	8	8	8	8

Fig. S21 MIC of different formulations in vitro. a, MIC of ceftazidime (CAZ), CAZ@BiNCs, M-MFL@CAZ and M-MFL@CB, respectively. b, MIC of ciprofloxacin (CIP), CIP@BiNCs, M-MFL@CIP and M-MFL@CIPB, respectively. c, MIC of colistin (COL), COL@BiNCs, M-MFL@COL and M-MFL@COLB, respectively.

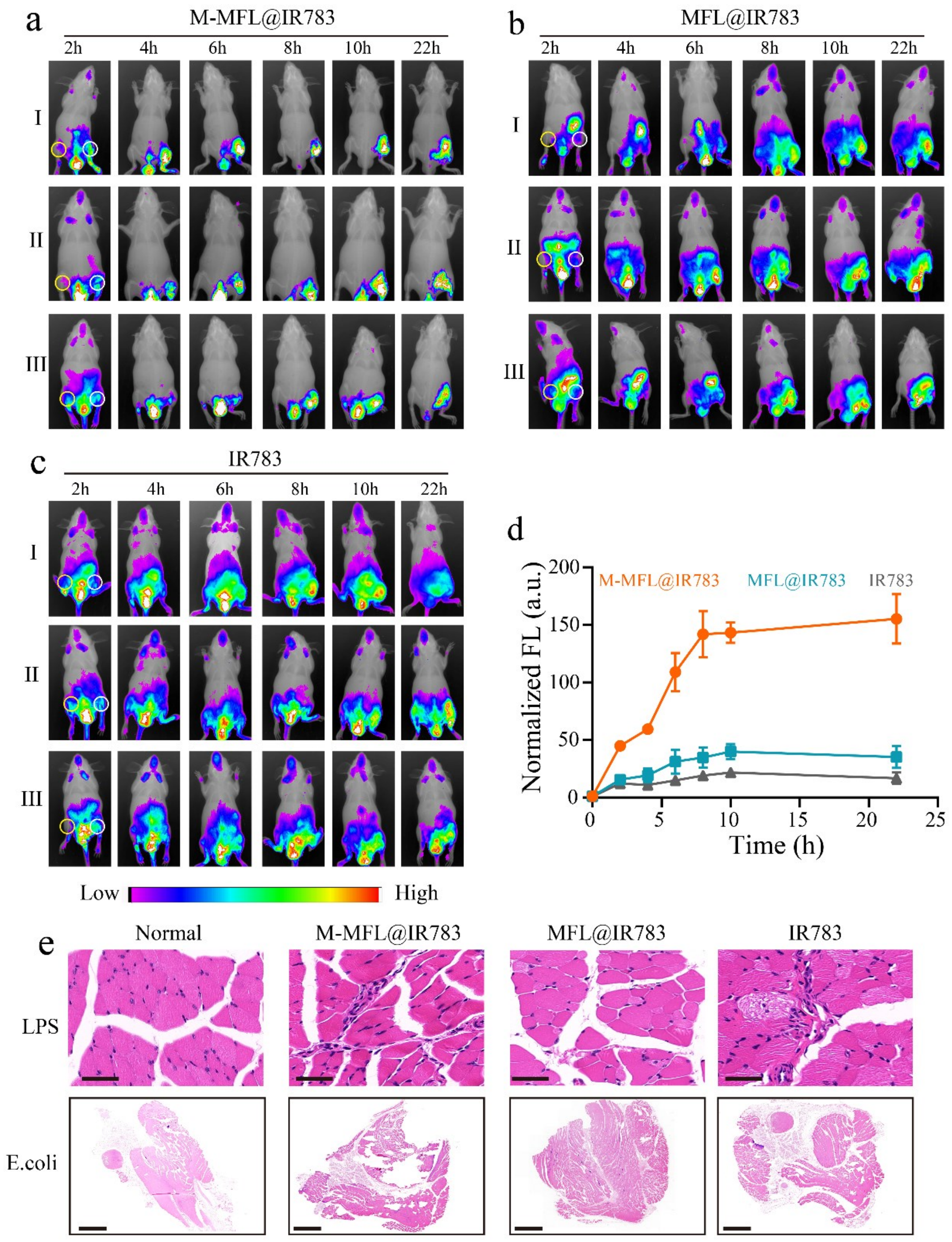

Fig. S22 Visualization of nanoparticles distribution in vivo. a, b c, d, NIR FL images of NDM-EC1349 infected mice model after tail vein injected with M-MFL@IR783 (a), MFL@IR783 (b), or IR780 (c), respectively and quantitative analysis of FL intensity (d) (n=3). The result showed that M-MFL@IR783 could more effectively and specifically accumulate into the bacterial infectious site compared with MFL@IR783 and IR783, respectively. e HE staining of LPS-infected (upper) and Gram staining of *E.coli*-infected

thigh muscles (lower), which demonstrated the successful preparation of mice model of dual infection with LPS and *E.coli*. Scale bar, 200 μm. Data are presented as mean values ± SD, n=3 biologically independent samples.

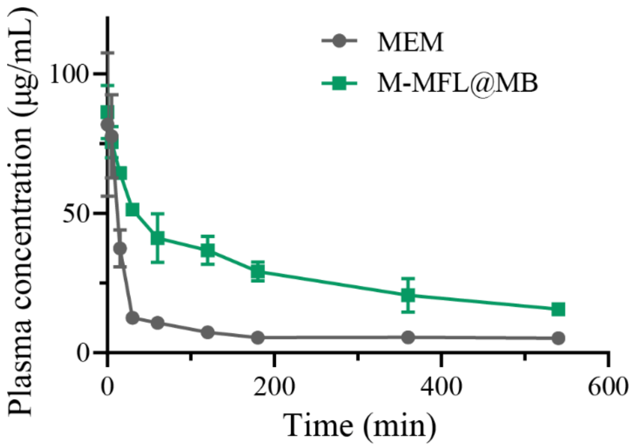

Fig. S23 The pharmacokinetic curve. The plasma MEM concentration was detected after a single tail vein injection of M-MFL@MB and MEM, respectively (n=3). M-MFL@MB administration led to higher drug concentrations in plasma compared with MEM at these indicating time points, revealing that M-MFL@MB had a more significant increase in blood circulation time. Data are presented as mean values ± SD, n=3 biologically independent samples.

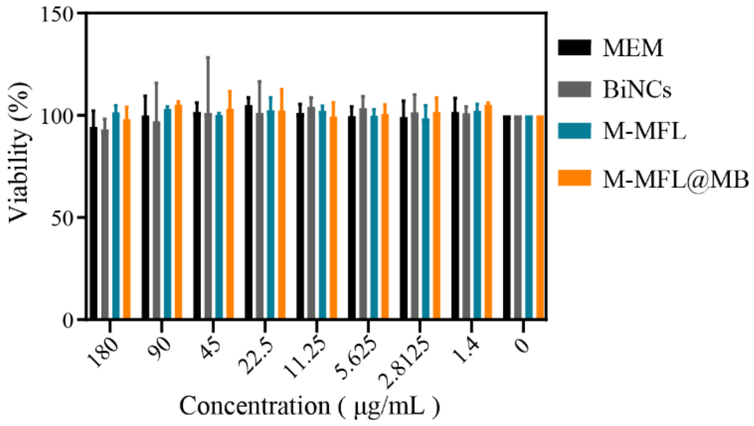

Fig. S24 Cytotoxicity assessment. The cytotoxicity assay was conducted by a standard CCK8 procedure after cells were incubated with different concentrations of nanoparticles for 24 h (n=6). due to the liver is the major metabolic organ, normal hepatocytes (HL-7702) are used as model cells. Benefiting from the high safety of each component, including BiNCs and M-MFL, M-MFL@MB exhibited excellent cytocompatibility. Data are presented as mean values ± SD, n=6 biologically independent samples.

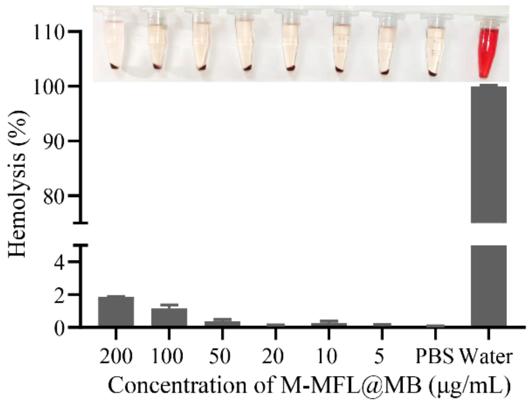

Fig. S25 Biohemocompatibility analysis. Hemolysis assays of treated with different concentrations of M-MFL@MB were conducted (n=6). The positive group is red blood cells swollen with ultra-pure water. M-MFL@MB formulation cannot cause hemolysis. Data are presented as mean values ± SD, n=6 biologically independent samples.

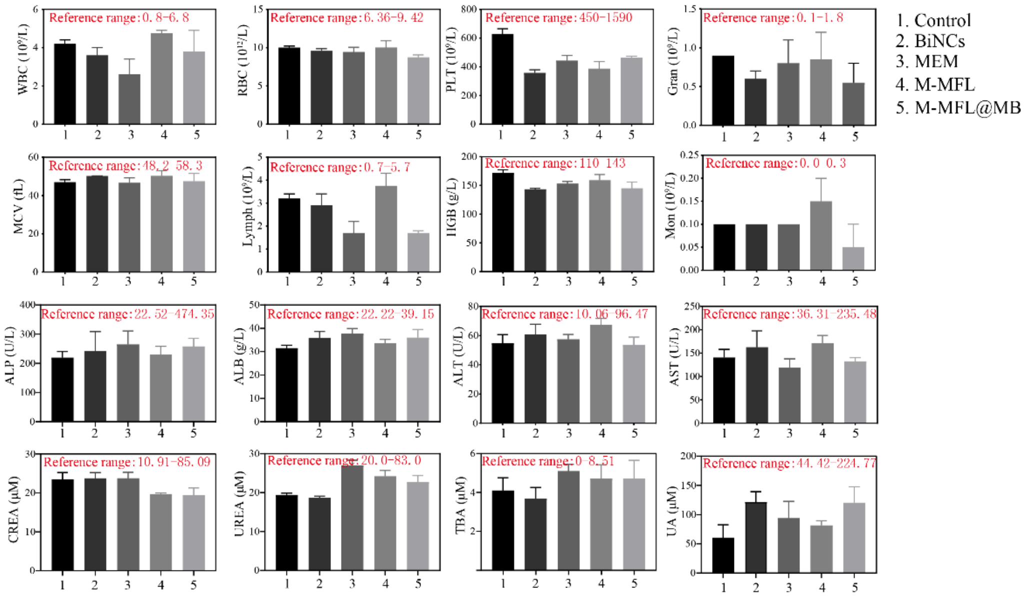

Fig. S26 Blood routine and blood biochemical analysis. Blood routine and blood biochemical indexes of the mice were determined after treatment with BiNCs, MEM, M-MFL, and M-MFL@MB, respectively (n=3). These indexes of blood routine and blood biochemistry after the tail vein injected with these nanoparticles fluctuated within normal limits. Data are presented as mean values ± SD, n=6 biologically independent samples.

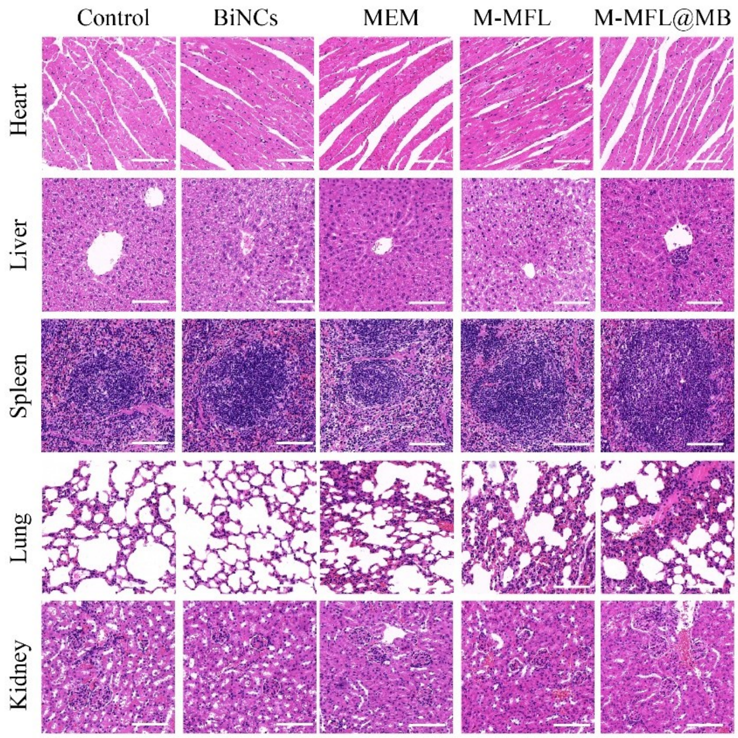

Fig. S27 Histocompatibility analysis. Hematoxylin and eosin (H&E) staining were conducted for visualizing the changes in major organs including heart, liver, spleen, lung, and kidney from healthy mice after tail vein injected with different formulations, including BiNCs, MEM, M-MFL and M-MFL@MB (Scar bar, 200 μm). M-MFL@MB has good histocompatibility.

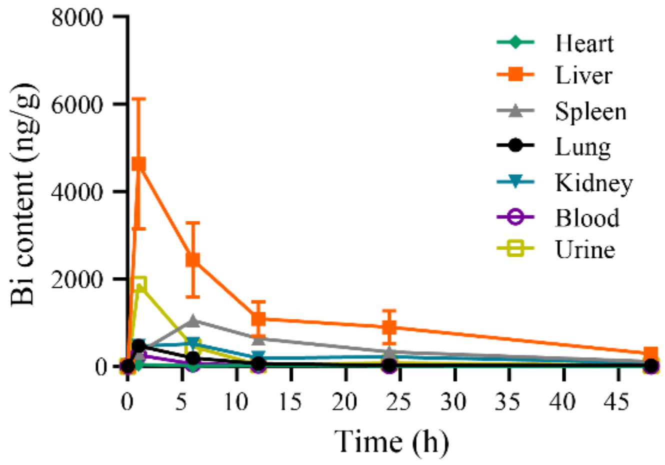

Fig. S28 In vivo metabolism of bismuth. ICP-MS assay was conducted for assessing the bismuth amounts of different organs or body fluids in healthy mice after tail vein was injected with M-MDL@MB (n=3). The liver and spleen are dominant organs for the accumulation and metabolism of bismuth, which may be mainly due to RES absorption. Data are presented as mean values ± SD, n=3 biologically independent samples.

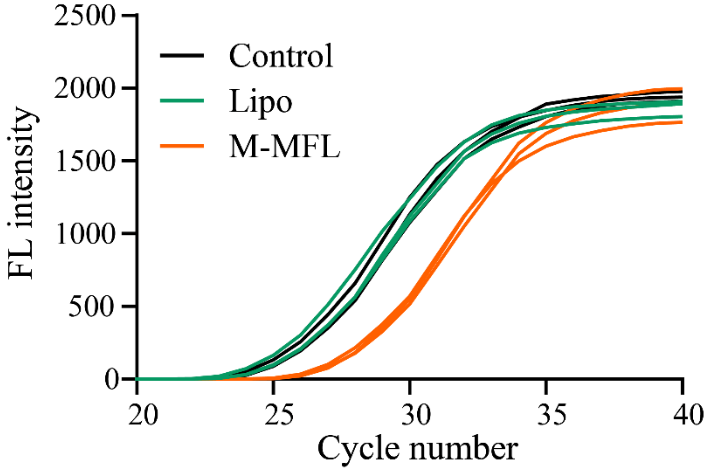

Fig. S29 Absolute quantification of blaNDM-1 in EVs. Fluorescence amplification curve in outer membrane vesicles (OMVs) extracted from *E.coli* incubated with Lipo and M-MFL, respectively. The OMVs extracted from untreated *E.coli* were used as control. n=3. Real-time PCR results reflected that M-MFL-treated *E.coli* secret the OMVs containing lower levels of blaNDM-1 gene compared with lipo or untreated *E.coli*, respectively.

Table S1 : Bacterial information

Isolates	Clinical features			NDM subtype	STs	MICs (μg/mL) for meropenem
	Age(yr)/ Sex	Specimen	Outcome			
EC-03	58 /male	Blood	Death	NDM-1	ST361	32
EC-13-22	41/female	Peritoneal drainage fluid	Discharge	NDM-1	ST361	16
EC13-33	68 /male	Blood	Discharge	NDM-1	ST540	4
EC-13-49	78/female	Urine	Discharge	NDM-1	ST167	16
EC-15-3	53/female	Urine	Discharge	NDM-1	ST6388	64
EC-16-7	52/female	Urine	Discharge	NDM-1	ST167	32
